# Natural Products of Freshwater Fungi from a Chemical and Bioactive Perspective

**DOI:** 10.3390/jof12040263

**Published:** 2026-04-03

**Authors:** Xiao-Jie Chen, Guo-Jun Zhou, Qian-Hong Yan, Xi Tan, Feng Xu, Fu-Ji Qian, Xu-Hang Fan, Bei Jiang, Cai-Juan Zheng, Hai-Feng Li, Kai-Ling Wang

**Affiliations:** 1 Yunnan Key Laboratory of Screening and Research on Anti-Pathogenic Plant Resources from Western Yunnan, Institute of Materia Medica, College of Pharmacy, Dali University, Dali 671000, China; cxj20182000@163.com (X.-J.C.); 15687617036@163.com (Q.-H.Y.); 17680419639@163.com (X.T.); 19187041748@163.com (F.X.); a18487642574@163.com (F.-J.Q.); dalinorthjiang@163.com (B.J.); 2 Key Laboratory of Tropical Medicinal Resource Chemistry of Ministry of Education, College of Chemistry and Chemical Engineering, Hainan Normal University, Haikou 571158, China; yaoxuewsw@163.com (G.-J.Z.); caijuan2002@163.com (C.-J.Z.); 3 Queen Mary School, Nanchang University, Nanchang 330031, China; fxh18008721121@163.com

**Keywords:** freshwater fungi, natural products, bioactivity, cytotoxicity, antimicrobial activity

## Abstract

Freshwater fungi have attracted considerable attention as a potential source of lead compounds with diverse and novel chemical structures and biological activities in drug discovery. This review summarizes 307 natural products of freshwater fungi from 1988 to the end of October 2025. These compounds are categorized into fourteen structural types, including fatty acids and their lactones (compounds **1**–**18**), furans and furanones (compounds **19**–**31**), pyrans and pyranones (compounds **32**–**109**), benzoquinones, phenols and phenolic acids (compounds **110**–**141**), naphthalenes and naphthalenones (compounds **142**–**192**), authraquinones and xanthones (compounds **193**–**211**, depsidones (compounds **212**–**217**), macrolides (compounds **218**–**234**), polyesters (compounds **235**–**237**), alkaloids (compounds **238**–**251**), peptides (compounds **252**–**280**), terpenoids (compounds **281**–**300**), steroids (compounds **301** and **302**), and other compounds (compounds **303**–**307**). Some of them displayed promising biological activity, mainly comprising antibacterial, cytotoxic, and nematicidal activities. The preliminary analysis of the Structure––Activity Relationship (SAR) of important compounds is also discussed. In the last section, current challenges and prospective research perspectives are briefly proposed based on opinions from previous reviews. This review would contribute to the understanding of the utilization and development of natural products derived from freshwater fungi as potent medical resources in the future.

## 1. Introduction

Fungi are widely recognized as a significant source of microbial natural products [1]. Some fungal strains can produce secondary metabolites with diverse chemical structures [2]. Many of these compounds exhibit various bioactivities, especially in antimicrobial and cytotoxic properties [3,4]. It is known that fungi spread throughout nearly all environments, including freshwater ecosystems. Freshwater habitats are made up of various submerged substrates or sediments, such as leaves, roots, wood, silt, mud, and other organic debris that freshwater microbes can feed on [5]. Therefore, freshwater fungi are described as those whose entire or part of their life cycle is dependent on freshwater settings, comprising species that function in water and terrestrial fungi that release spores into water [5]. To resist attacks from competitors and predators, many freshwater fungi have evolved chemical defense strategies, which are crucial for their survival and resource acquisition in the aquatic environment [6].

To date, more than 95% of fungal taxa remain undescribed, of which reported freshwater–derived fungi account for only approximately 3% [7,8]. The genomic studies of aquatic fungi suggest that some fungal strains should have high metabolite production capabilities, yet the expression of their biosynthetic gene clusters is largely silenced [9], which further indicates that the current utilization and development of natural products from freshwater fungi is simply the tip of the iceberg. Although freshwater microorganisms have long been discovered from submerged substrates in the Lake District, England [5], it was not until 1988 that the first report describing a new antibiotic quinaphthin produced by the freshwater fungus *Helicoon richonis* was published in the Journal of Transactions of the British Mycological Society [10]. Since then, more and more functional natural products derived from freshwater microbes gradually attracted increasing attention. To our best knowledge, there are only two reviews that have reviewed the research advances of freshwater fungi at different stages, mainly focusing on species and chemical diversity of aquatic fungi [5,11]. However, with the development of separation technology of natural products, more and more freshwater fungi and their secondary metabolites have been discovered. Actually, some freshwater fungi–sourced natural products have displayed potential for the development of biologically active agents [12,13,14,15,16], which needs to be summarized more systematically from chemical and bioactive profiling of secondary metabolites of freshwater fungi.

**Figure 1:**
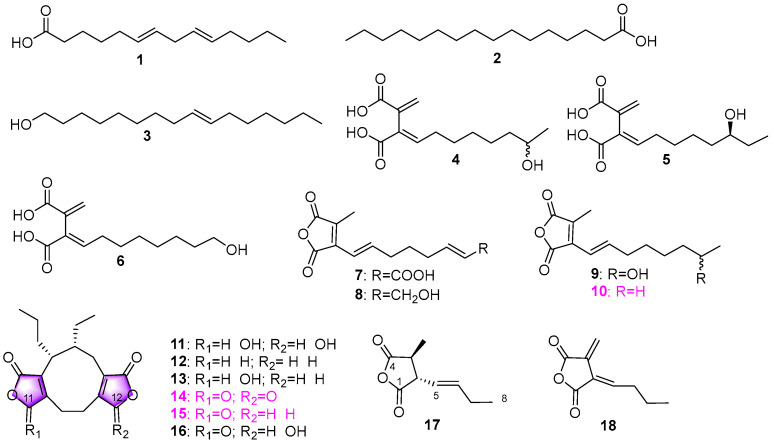
Chemical structures of compound **1** from *Lindgomyces angustiascus*, **2** and **3** from *Minutisphaera fimbriatispora*, **4**–**10** from *Tricladium castaneicola*, and **11**–**18** from *Wicklowia aquatica.* The purple highlighted indicates that the presence of the C–11, 12 carbonyl group of compounds **11**–**16** is related to antifungal activity.

**Figure 2:**
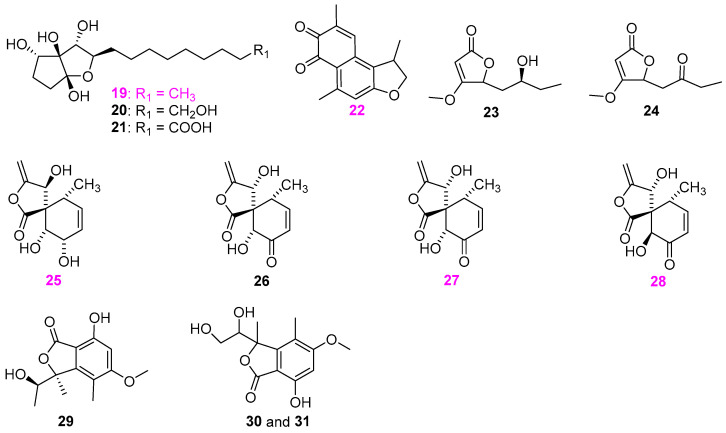
Chemical structures of compounds **19**–**21** from *Helicodendron giganteum*, **22** from *Chaetosphaeriaceae* sp., **23** and **24** from *Annulatascus triseptatus*, **25**–**28** from *Massarina tunicata*, **29** from *Paraphoma radicina*, and **30** and **31** from two fungal strains (possibly *Entrophospora* sp. and *Phaeosphaeria* sp.).

**Figure 3:**
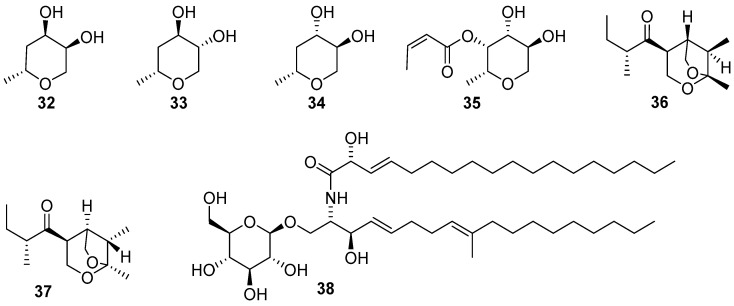
Chemical structures of compounds **32**–**35** from *Ophioceras venezuelense*, and **36**–**38** from an unidentified freshwater fungus YMF 1.01029.

**Figure 4:**
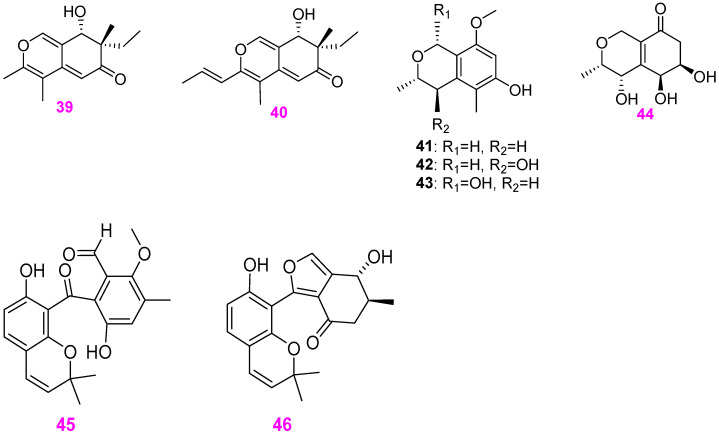
Chemical structures of compounds **39** and **40** from *Pseudohalonectria adversaria*, **41**–**44** from *Penicillium* sp., and **45** and **46** from *Massarina tunicata*.

**Figure 5:**
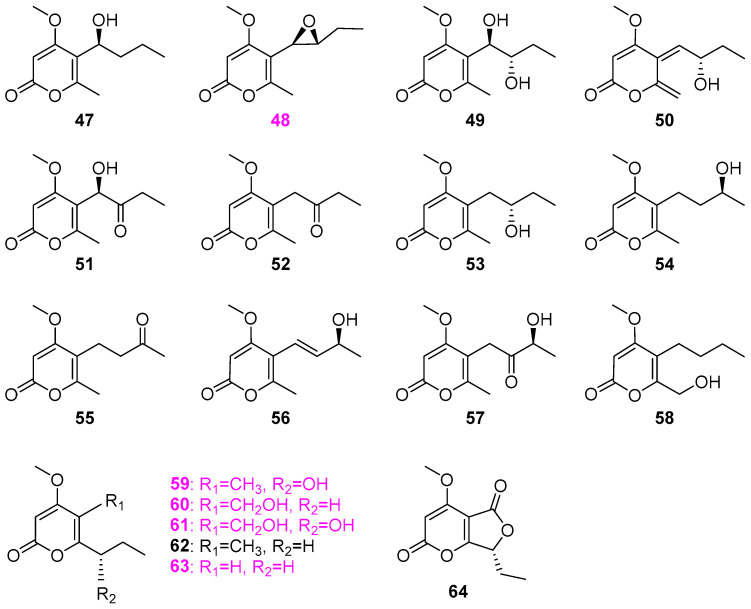
Chemical structures of compounds **47**–**58** from *Delitschia* sp., and **59**–**64** from *Annulatascus triseptatus*.

**Figure 6:**
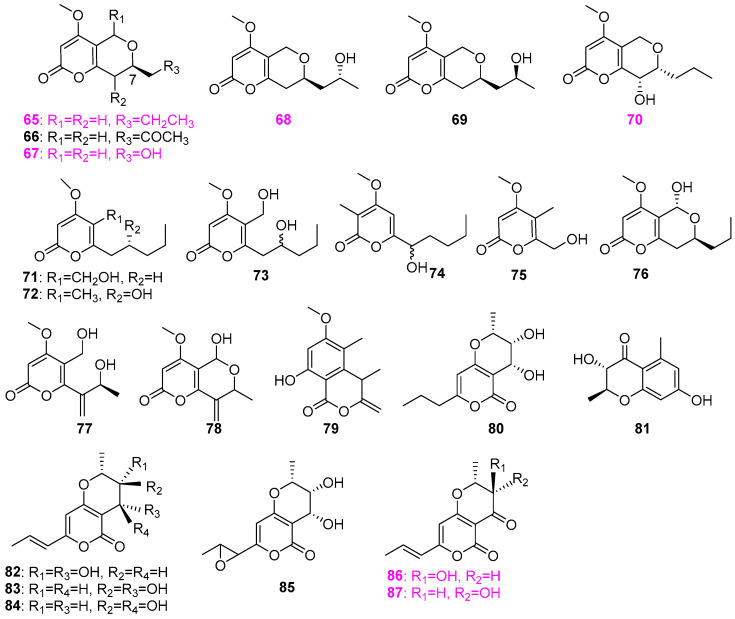
Chemical structures of compounds **65**–**76** from *Clohesyomyces* sp. and *C. aquaticus*, **77**–**79** from a Glomeromycete (possibly *Entrophospora* sp.) and a Dothideomycete (possibly *Phaeosphaeria* sp.), and **80**–**87** from *Xylomyces chlamydosporus*.

**Figure 7:**
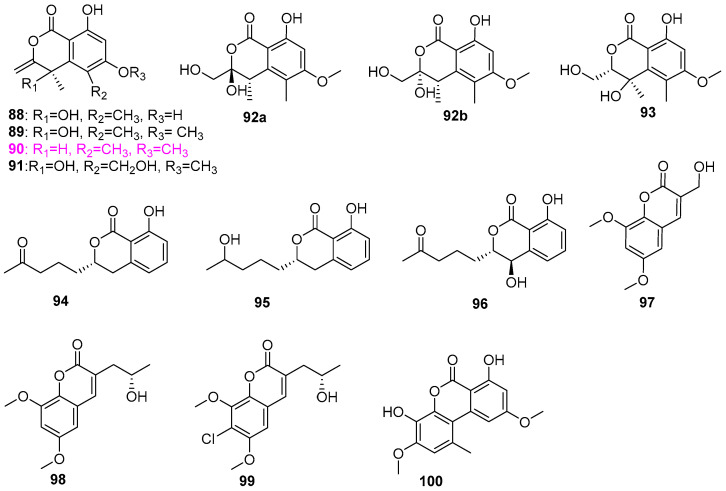
Chemical structures of compounds **88**–**93** from *Paraphoma radicina*, and **94**–**100** from *Talaromyces amestolkiae*.

**Figure 8:**
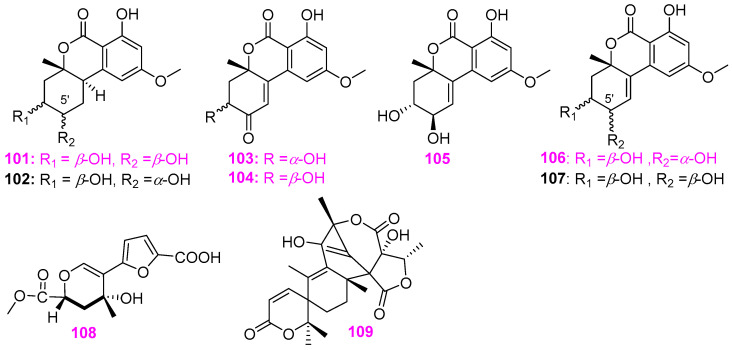
Chemical structures of compounds **101**–**107** from an unidentified aquatic fungal strain A–00471, and **108** and **109** from *Penicillium* sp.

**Figure 9:**
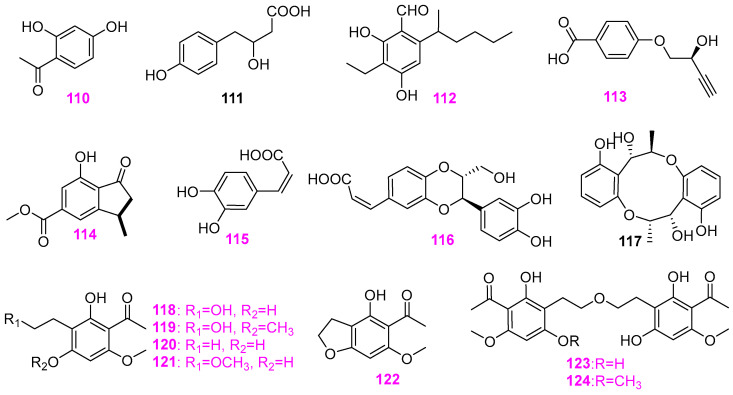
Chemical structures of compound **110** from *Camposporium quercicola*, **111** from *Annulatascus triseptatus*, **112** from *Anguillospora longissima*, **113** from *Massarina tunicate*, **114** from *Penicillium* sp., **115**–**117** from *Ophioceras dolichostomum*, and **118**–**124** from *Lindgomyces madisonensis*.

**Figure 10:**
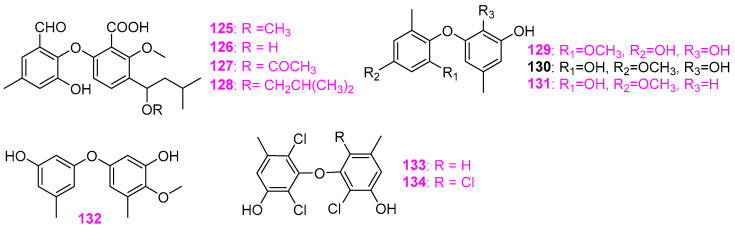
Chemical structures of compounds **125** from *Dendrospora tenella* and *Camposporium quercicola*, **126**–**128** from *Dendrospora tenella*, **129**–**131** from *Phoma* sp., **132** from *Camposporium quercicola*, and **133** and **134** from *Kirsrhsteiniotbelia* sp.

**Figure 11:**
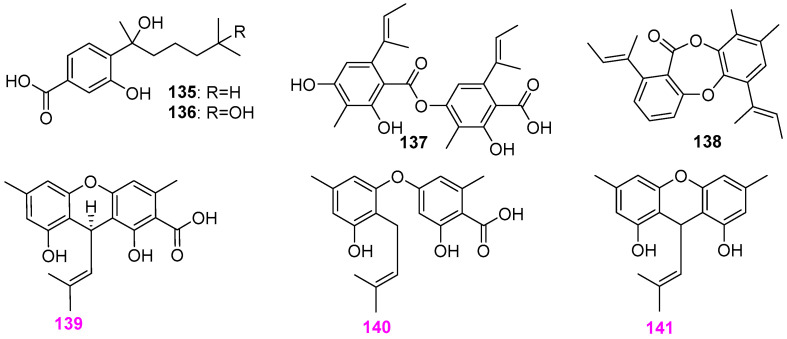
Chemical structures of compounds **135**–**138** from *Wicklowia aquatic*, and **139**–**141** from *Helotiales* sp.

**Figure 12:**
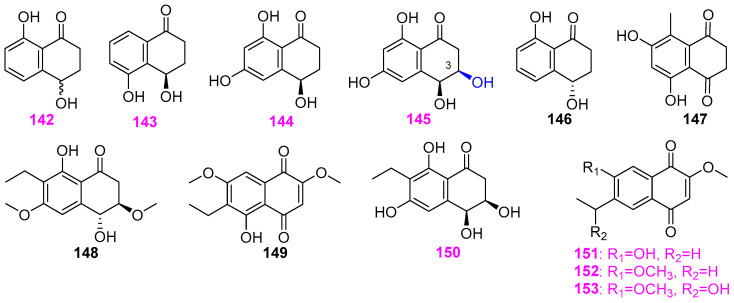
Chemical structures of compounds **142**–**145** from *Caryospora callicarpa*, **146** and **147** from *Paraphoma radicina*, **148** and **149** from *Delitschia* sp., and **150**–**153** from *Delitschia corticola*. The blue markings indicate active key functional groups.

**Figure 13:**
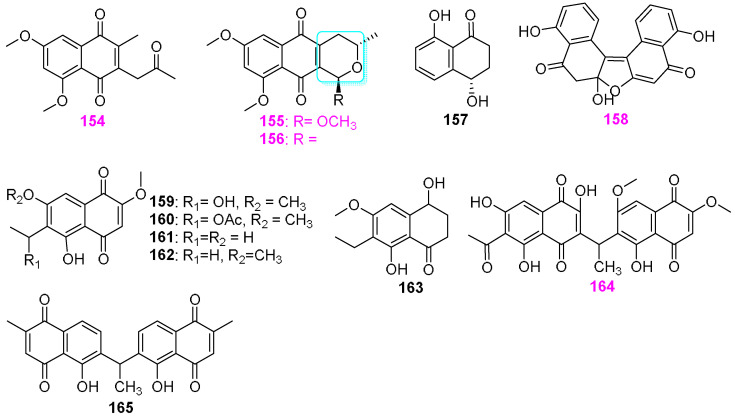
Chemical structures of compounds **154**–**156** from *Astrophaeriella papuana*, **157** from *Minutisphaera parafimbriatispora* and *M. aspera*, **158** from *M. parafimbriatispora*, and **159**–**165** from *Kirschsteiniothelia funicularia.* The part circled in lake blue is a rare structure.

**Figure 14:**
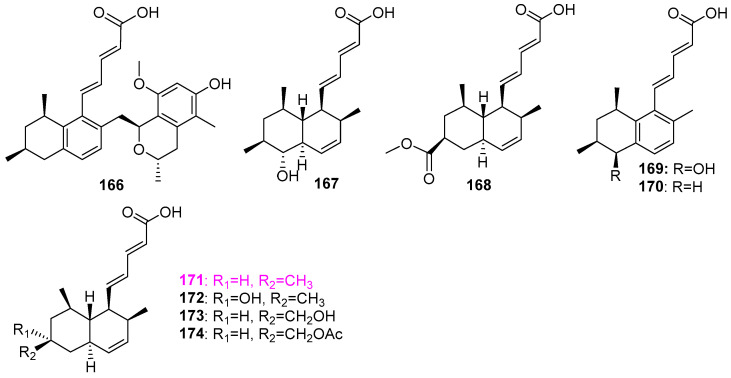
Chemical structures of compounds **166**–**174** from *Penicillium* sp.

**Figure 15:**
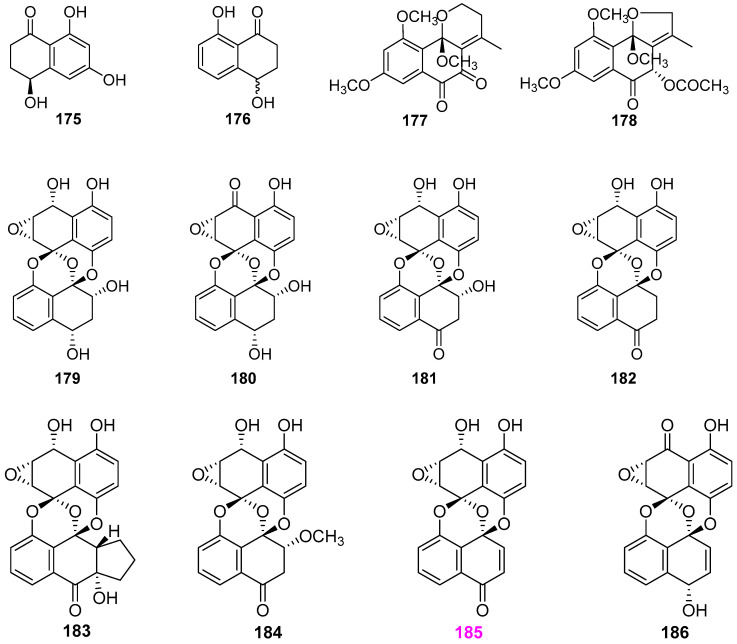
Chemical structures of compounds **175**–**186** from an unidentified fungus YMF 1.01029.

**Figure 16:**
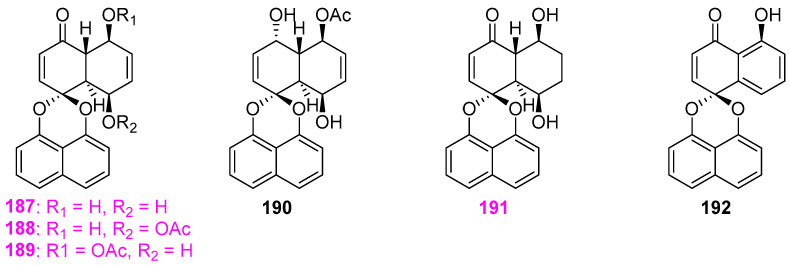
Chemical structures of compounds **187**–**192** from *Decaisnella thyridioides*.

**Figure 17:**
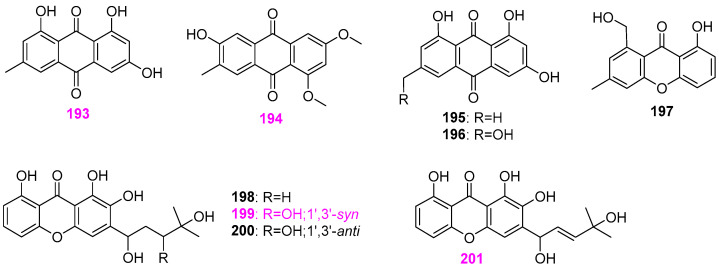
Chemical structures of compounds **193** from *Penicillium* sp., 194 from *Astrophaeriella papuana*, **195**–**197** from *Chaetomium* sp., and **198**–**201** from *Chaetosphaeriaceae* sp.

**Figure 18:**
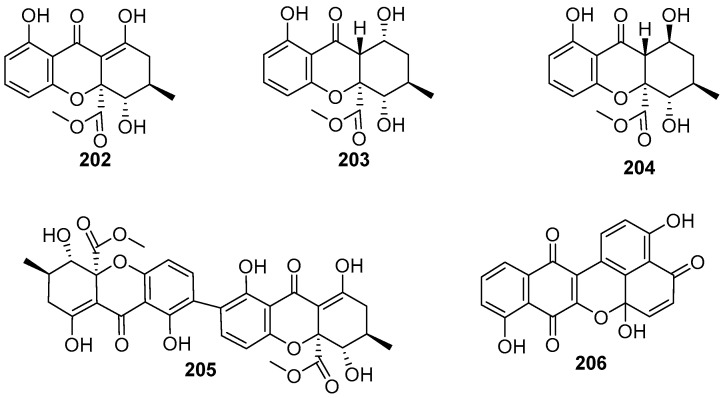
Chemical structures of compounds **202**–**205** from *Clohesyomyces* sp., and **206** from *Helicoon richonis*.

**Figure 19:**
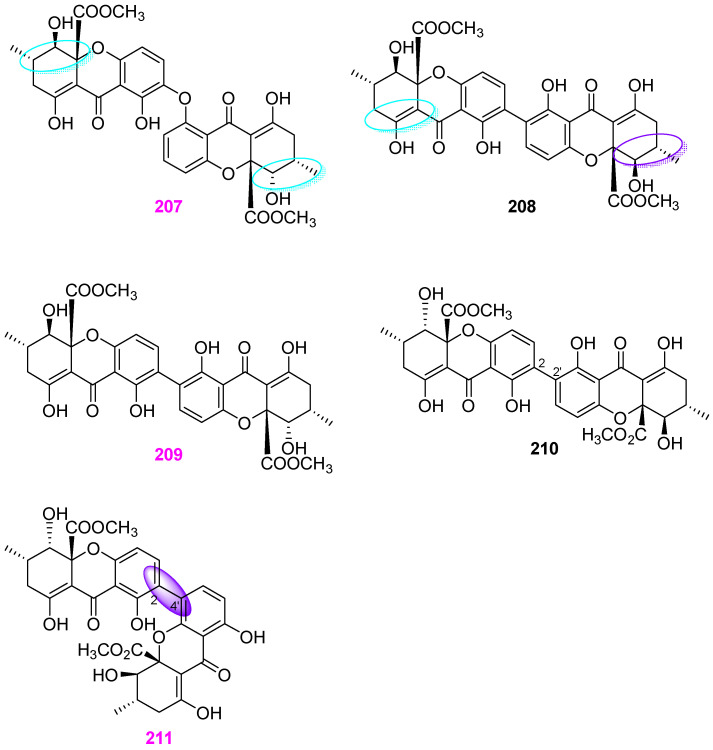
Chemical structures of compounds **207**–**211** from *Aspergillus* sp. The part circled in color indicates that structures feature a heterodimeric tetrahydroxanthone skeleton.

**Figure 20:**
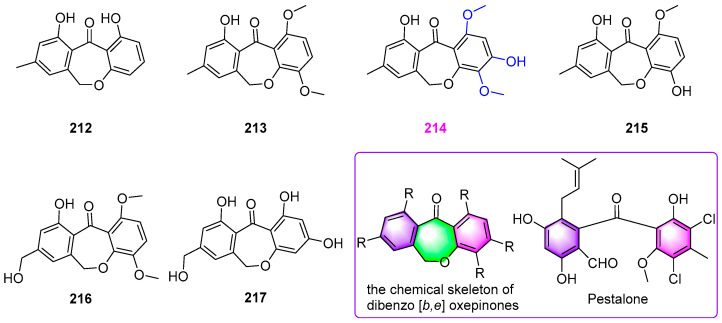
Chemical structures of compounds **212**–**217** from *Chaetomium* sp. and pestalone. The blue markings indicate hydrophobicity of the dibenzo [*b*,*e*] oxepin core in compound **214** related to the cytotoxic activity, the colored entities on the left indicate the rarity of the structure, while those on the right denote similar structures with potential antibiotic properties.

**Figure 21:**
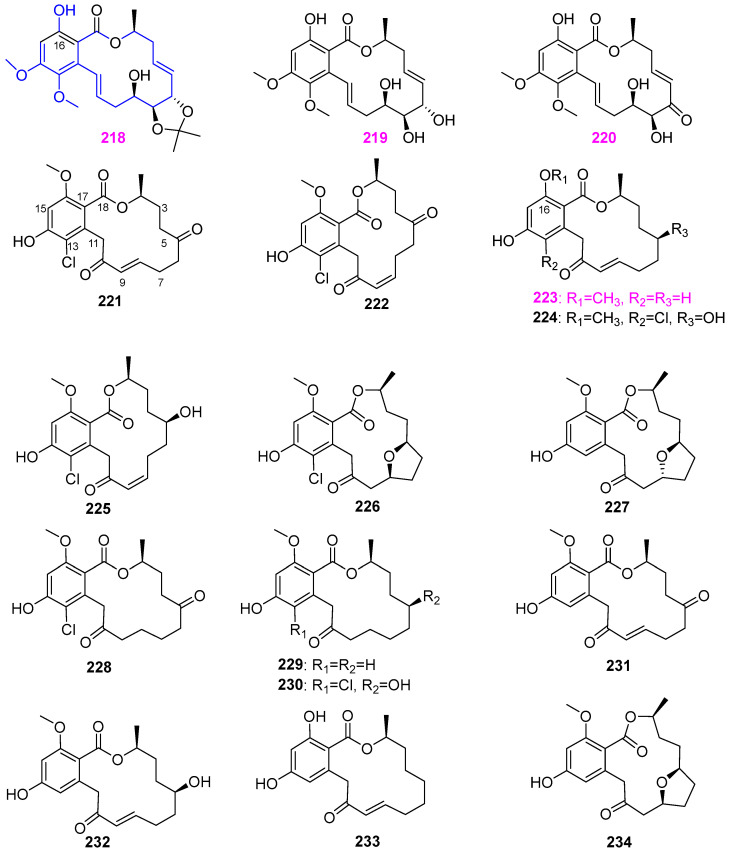
Chemical structures of compounds **218**–**220** from *Caryospora callicarpa*, and **221**–**234** from *Halenospora* sp. The blue mark indicates a rare structure.

Herein, this review summarizes 307 secondary metabolites from freshwater fungi between 1988 and 2025. These fungal strains were mainly collected from the United States of America (USA) and China, and their natural products are sequentially introduced based on parent nucleus, including fatty acids and their lactones **1**–**18**, furans and furanones **19**–**31**, pyrans and pyranones **32**–**109**, benzoquinones, phenols and phenolic acids **110**–**141**, naphthalenes and naphthalenones **142**–**192**, authraquinones and xanthones **193**–**211**, depsidones **212**–**217**, macrolides **218**–**234**, polyesters **235**–**237**, alkaloids **238**–**251**, peptides **252**–**280**, terpenoids **281**–**300**, steroids **301** and **302**, and other five compounds **303**–**307**. The biological activity and Structure–Activity Relationship (SAR) of important compounds are simultaneously discussed. In Figures 1–31 with structural formulas, the compounds with excellent bioactivity were highlighted uniformly in rose red, and some novel compounds with rare chemical skeletons were also marked by different colors. It is worth noting that in the conclusion section, we conducted a statistical analysis of the classification, biological activity, and freshwater fungal sources of 307 compounds, presenting the research findings in chart and graph formats (Figures 32 and 33). Finally, based on the existing research findings, the potentials and challenges are briefly proposed with respect to the development and utilization of secondary metabolites from freshwater fungi as bioactive agents.

## 2. Natural Products from Freshwater Fungi and Their Bioactivities

### 2.1. Fatty Acids and Their Lactones

The lignicolous fungus *Lindgomyces angustiascus* (G202–1) was proposed as a new species isolated from submerged wood in lotic and lentic habitats from Florida, North Carolina, and Wisconsin, USA [17]. Chemical analysis of secondary metabolites production of the fungal strain G202–1 yielded a fatty acid, 6*E*,9*E*–octadecadienoic acid (**1**) (Figure 1). Additionally, palmitic acid (**2**) and (*E*)–hexadec–9–en–1–ol (**3**) (Figure 1) were isolated from the aquatic fungus *Minutisphaera fimbriatispora* distributed in both Aomori (Japan) and North Carolina (USA) [18]. However, the biological activity of all three of the above compounds was not mentioned in the related literature [17]. The aquatic hyphomycete *Tricladium castaneicola* AJ117567 was isolated from leaves collected from a mountain stream in Hakone, Kanagawa, Japan [19]. A total of seven novel compounds, including three tricladic acids A–C (**4**–**6**) and four maleic anhydride derivatives tricladolides A–D (**7**–**10**) (Figure 1), were obtained. Among them, tricladolides A–D (**7**–**10**) could selectively inhibit the growth of *phytophthora* fungal strains but were inactive against yeasts and bacteria. Specifically, tricladolide D (**10**), possessing a lipophilic alkyl chain (as shown in Figure 1), showed the highest antifungal activity against *Phytophthora* with a 5 mm inhibitory zone at 100 µg/disk. Meanwhile, succinic acid–based tricladic acids B (**5**) and C (**6**) are still active despite hydroxylation on the alkyl chain, which suggests that the succinic acid portion should be more effective than maleic anhydride for increasing anti–phytophthora activity of these compounds. In addition, tricladolide D (**10**), as the most lipophilic compound, showed the highest inhibitory activity against the B16 melanoma cells with an IC_50_ value of 80 µmol/L. The fungus *Wicklowia aquatica* F76–1, associated with herbaceous material collected from a backwater swamp in the Ocala National Forest in northern Florida, was found to produce three new nonadride analogs: tetrahydroepiheveadride (**11**), dideoxoepiheveadride (**12**), and deoxodihydroepiheveadride (**13**), together with three known compounds **14**–**16**, and two new compounds, waquafranones A (**17**) and B (**18**) (Figure 1) [20]. It was proposed that the nonadrides might be biosynthesized via dimerization of C–9 units, which indicated that the C–9 compounds **17** and **18** could be considered as the monomeric precursors of nonadrides. From these eight compounds, epiheveadride (**14**), deoxoepiheveadride (**15**), and dihydroepiheveadride (**16**) were active toward *Aspergillus flavus* NRRL6541 and *Fusarium verticillioides* NRRL2545. The analysis of SAR indicated that the presence of the carbonyl group at C–11, 12 (Figure 1) might be related to the intensity of antifungal activity.

### 2.2. Furan and Furanone Derivatives

Three new compounds, heliconols A–C (**19**–**21**) (Figure 2), were isolated from the aquatic fungus *Helicodendron giganteum* (CS988–1B) derived from submerged wood in Alaska, USA [21]. Among them, only heliconol A (**19**) exhibited antimicrobial activity against *Fusarium Verticillioides* NRRL 25457, *Candida albicans* ATCC 14053, *Staphylococcus aureus* ATCC 29213, and *Bacillus subtilis* ATCC 6051 in agar disk diffusion assays. After 48 h of treatment with 100 µg/disk, this compound produced inhibition zones of 18, 23, and 35 mm against *C. albicans* ATCC 14053, *S. aureus* ATCC 29213, and *B. subtilis* ATCC 6051, respectively. Meanwhile, compound **19** was also capable of inhibiting the growth of *F. Verticillioides* NRRL 25457 with a 15 mm zone of inhibition at 200 µg/disk. A known polyketide metabolite, mansonone D (**22**) (Figure 2), was isolated from the freshwater fungus *Chaetosphaeriaceae* sp. BCC 28210 is associated with a submerged wood collected from Tai Rom Yen National Park (Surat Thani province, Thailand) [22]. This compound exhibited antimalarial activity against *Plasmodium falciparum* K1, antifungal activity against *Candida albican*, and cytotoxicity against Vero (African green monkey kidney fibroblast) cells with respective IC_50_ values of 0.55, 1.95, and 1.97 µg/mL, but no activity against *Bacillus cereus.* The freshwater fungus *Annulatascus triseptatus* A–353–1B, derived from submerged woody debris in Shaker Pond, Alfred, Maine, USA, was identified as the first member of a new genus and species [23]. It was demonstrated that the fungal strain A–353–1B could produce a series of furanone and pyranone metabolites, among which two new furanones were named as annularins G (**23**) and H (**24**) (Figure 2) [24]. Both compounds showed no antibacterial activity against *Bacillus subtilis* (ATCC 6051) and *Staphylococcus aureus* (ATCC 29213) (200 µg/disk) in the standard disk assay. Four new rosigenin analogs, massarigenins A–D (**25**–**28**) (Figure 2), were obtained from the aquatic fungus *Massarina tunicata* A–25–1 [25]. The structure of massarigenin A (**25**) was verified via X–ray crystallograph. At 200 µg/disk, except for compound **26**, all the compounds displayed antibacterial activity against *Bacillus subtilis* (ATCC 6051) with zones of inhibition of 17, 23, 11, 9, and 14 mm, respectively. A isobenzofuranone (**29**) (Figure 2) was obtained from the fungus *Paraphoma radicina* G104 associated with submerged wood in a freshwater lake of Greensboro, North Carolina, USA, and was inactive against *Staphylococcus aureus*, *S. aureus*, *S. cerevisiae*, *Escherichia coli*, *Mycobacterium smegmatis*, and *Aspergillus niger* at the concentration of 153 μg/mL [26]. Two of the fungal strains, a Glomeromycete (possibly *Entrophospora* sp. with GenBank accession no. JQ958304) and a Dothideomycete (possibly *Phaeosphaeria* sp. with GenBank accession no. JQ958305), were isolated from a naturally occurring microbial mat in an iron–rich natural spring that feeds into Clear Creek near Golden, CO, USA [27]. The scale–up chemical investigation of these two fungi resulted in a new 1:1 mixture of structurally related metabolite clearanol E (**30** and **31**) (Figure 2), none of which were active against the bacteria *Staphylococcus aureus* (ATCC 700787), and *Klebsiella pneumoniae* (ATCC 51503), and the fungi polyene–resistant *Candida albicans* (ATCC 38245) and *Aspergillus fumigatus* (FGSC A1100).

### 2.3. Pyran and Pyranone Derivatives

The pyrenomycete fungus *Ophioceras venezuelense* A447–1B occuring on submerged wood and herbaceous debris habited in freshwater in Venezuela and Costa Rica was fermented on rice [28]. And then the solid–substrate cultures were extracted by ethyl acetate (EtOAc), yielding four new tetrahydropyran ophiocerins A–D (**32–35**) (Figure 3). These four compounds exhibited neither antibacterial (*Staphylococcus aureus* and *Escherichia coli*) nor antifungal (*Candida albicans*) activity at 200 µg/disk. Dong et al. [29] obtained two novel diastereoisomeric bicyclic ketals colomitides **36** and **37** (Figure 3) with an unusual 2,7–dioxabicyclo[3.2.1]octane ring system, and a pyran derivative compound **38** (Figure 3) from an unidentified freshwater fungus YMF 1.01029, which was isolated from the decaying branches of an unidentified tree near Lake Fuxian in Yunnan province, China [29]. This is the first report of colomitides derived from a fungal microorganism. Such compounds are rare in nature. The dioxabicyclo[3.2.1]octane metabolites **36** and **37** possess the closest structures to the insect pheromones, which might be of significant biocontrol importance. Antimicrobial activity of all three compounds was tested against four bacterial pathogens of *Bacillus subtilis* YMF 3.19, *B. laterosporus* YMF 3.08, *Staphylococcus aureus* YMF 3.17, and *Escherichia coli* YMF 3.16, and seven pathogenic fungi of *Bipolaris maydis* YMF 1.2094, *Botrytis* sp. YMF 1.02079, *Cochliobolus sativus* YMF 1.2088, *Colletotrichum* sp. YMF 1.02099, *Fusarium verticillioides* YMF 1.2076, *Rhizoctonia solani* YMF 1.02096, and *Sclerotinia sclorotiorum* YMF 1.02081. At 50 μg/disk, both colomitides **36** and **37** showed antibacterial activity against *B. subtilis* YMF 3.19, *B. laterosporus* YMF 3.08, and *S. aureus* YMF 3.17, with the diameter of inhibition areas ranging from 10 to 14 mm, and antifungal activity against *B. maydis* YMF 1.2094, *C. sativus* YMF 1.2088, and *F. verticillioides* YMF 1.2076 with zones of inhibition ranging from 8 to 13 mm. However, these two compounds were inactive against *E. coli* YMF 3.16, and compound **38** exhibited no activity against all the above eleven pathogens at the same concentration.

Dong et al. [30] also obtained two new mixed pyrone–quinone skeleton metabolites, pseudohalonectrins A (**39**) and B (**40**) (Figure 4), from the aquatic fungus *Pseudohalonectria adversaria* YMF1.01019 associated with submerged wood in Yunnan Province, China. This is the first report of natural products from any member of the genus *Pseudohalonectria*. Both compounds could kill pine wood nematode *Bursaphelenchus xylophilus* with respective mortality rates of 92.4% and 93.1% at 200 ppm after 36 h. Three known isochromans **41**–**43** (Figure 4) were isolated from the fungus *Penicillium* sp. (S1a1) derived from a freshwater sediment sample collected from Selinos River, Bergama– İzmir, in the western region of Turkey, at a depth of 1 m [31]. These three compounds were all inactive in the antagonistic activity against *Staphylococcus aureus* (ATCC 700699 and ATCC 29213) and *Mycobacterium tuberculosis* (H37Rv) and cytotoxicity activity against the mouse lymphoma (L5178Y) cell line. *Delitschia corticola* YMF 1.01111 from a submerged woody substrate collected in Yunna Province, China, was fermented via liquid shake culture. The EtOAc crude extract of the culture filtrate was isolated using column chromatography, resulting in the identification of a new metabolite (3S*,4S*,5S*,6R*)–4,5,6–trihydroxy–3–methyl–3,4,6,7–tetrahydro–1*H*–isochromen–8(5*H*)–one (**44**) (Figure 4) [12]. The new compound had potent antifungal activity against *Gibberella saubinetii* YMF 1.01989 and *Colletotrichum* sp. YMF 1.01994 at 50 μg/disk with 11 and 7 mm zones of inhibition, respectively. The freshwater–derived fungus *Massarina tunicata* A–25–1 was found to produce two new aromatic polyketide–derived secondary metabolites massarinins A (**45**) and B (**46**) (Figure 4) besides the furanones **25**–**28** mentioned above [25]. At 200 µg/disk, both compounds were each active against *Staphylococcus aureus* (ATCC 29213) and *Bacillus subtilis* (ATCC 6051), with the respective diameters of inhibition zones of 7, 6, 12, and 7 mm. However, none of the furanone and pyran derivatives produced by the fungal strain A–25–1 showed antifungal activity against *Candida albicans* (ATCC 14053), *Aspergillus flavus* (NRRL 6541), or *Fusarium verticillioides* (NRRL 25457) at the same level.

The fungus *Delitschia* sp. G858 isolated from submerged wood in LeBaup stream of Ariége, Rimont, France, was found to produce twelve compounds **47**–**58** (Figure 5), including eight new structurally related α–pyrone derivatives **47**–**53** and **57** [32]. The absolute configurations of each new compound were determined by modified Mosher’s ester method. Compounds **47**, **48**, **51–58** were evaluated for cytotoxicity against the African American prostate cancer cell line (E006AA–hT), among which only the new compound 1′,2′–epoxi–delitpyrone A (**48**) could effectively inhibit tumor cell proliferation at 40 µM. Five new pyranone metabolites, annularins A–E (**59**–**63**), a new fused α–pyrone–furanone metabolite, annularin F (**64**) (Figure 5), that was previously reported as a characteristic skeleton presenting in a natural product, along with two new furanones, **23** and **24** (Figure 2), mentioned above, were isolated from the organic extract of the freshwater fungus *Annulatascus triseptatus* A–353–1B [24]. Among compounds **59**–**64**, annularins A–C (**59**–**61**) and F (**63**) were active against *Bacillus subtilis* (ATCC 6051), each causing 8–10 mm zones of inhibition at 200 µg/disk, while compound annularin C (**61**) also displayed activity against *Staphylococcus aureus* (ATCC 29213), affording a 14 mm zone of inhibition.

Two fungal strains, G100 and G102, belonging to the genus *Clohesyomyces*, were isolated from wood submerged in the Lake Brandt watershed in Greensboro, USA [33]. Twelve *α*–pyrone derivatives **65**–**76** (Figure 6) were isolated from strain G102, among which compounds **65**, **67**, **70**, **71**, and **72** were also biosynthesized by strain G100. Although compounds **65** and **71**–**76** have no antimicrobial activity against an array of bacteria and fungi (*Staphylococcus aureus*, *Escherichia coli*, *Mycobacterium smegmatis*, *Candida albicans*, and *Aspergillus niger*), compounds **65**, **67**, **68**, and **70** exhibited varying degrees of inhibition for peptidyl–tRNA hydrolases (Pth1) in several bacterial species. Besides the mixture of furanones **30** and **31** (Figure 2), three new pyranones **77**–**79** (Figure 6) were also isolated from the fungal strains (possibly *Entrophospora* sp. and *Phaeosphaeria* sp.) and identified as clearanols A (**77**), B (**78**), and D (**79**) [27]. However, all three compounds showed no activity against *Staphylococcus aureus* (ATCC 700787), *Klebsiella pneumoniae* (ATCC 51503), polyene–resistant *Candida albicans* (ATCC 38245), and *Aspergillus fumigatus* (FGSC A1100). The fungus *Xylomyces chlamydosporus* H58–1 was isolated from submerged wood samples collected from a stream in the Great Smoky Mountains National Park, USA [13]. Solid–substrate fermentation cultures of *X. chlamydosporus* were extracted by EtOAc. Two new compounds [(2*S*, 3*S*, 4*S*)–3,4–dihydroxy–2–methyl–7–propyl–3,4–dihydro–2*H*,5*H*–pyrano[4,3–*b*]pyran–5–one (**80**) and (2*S*, 3*S*)–3,7–dihydroxy–2,5–dimethyl–chroman–4–one (**81**)], together with six known compounds **82**–**87** (Figure 6), were then obtained. The absolute configuration of compound **81** was established through modified Mosher’s method. Compounds radicinin (**86**) and 3–*epi*–radicinin (**87**) showed excellent antifungal activity against *Fusarium verticillioides* (NRRL 25457), producing 27 and 21 mm inhibitory zones at 100 μg/disk, which was comparable to that of the positive drug nystatin. However, all the compounds showed no activity against the fungus *Aspergillus flavus* (NRRL 6541).

The aquatic fungus *Paraphoma radicina* G104 was found to produce not only the aforementioned furanone compound **29** (Figure 2) but also six isochromenones (**88**–**93**) (Figure 7), including two new compounds of clearanols G (**91**) and F (**93**) [26]. All six pyranone compounds were also screened for antimicrobial activity against a panel of microorganisms mentioned above, among which only clearanol C (**90**) was active against *Staphylococcus aureus* at the concentration of 33 µg/mL. However, Gerea et al. [27] discovered that clearanol C (**90**) showed moderate inhibition on the fungal growth of *Candida albicans* and *Aspergillus fumigatus*, and could prevent the biofilm formation of *C. albicans* with MIC values of 101 ± 3 µmol/L, which is inconsistent with the results of the antifungal activity of this compound reported in El–Elimat et al. [26]. From the fungus *Talaromyces amestolkiae* (G173) associated with submerged wood collected in a small pond near Bur–Mil Park, Guilford County, North Carolina, USA, six compounds were isolated and identified as dihydroisocoumarins (**94**–**96**), coumarins (**97**–**99**), and dibenzo–a–pyrone (**100**) (Figure 7) [34]. Among them, compounds 7–chloropestalasin A (**99**) and 4–hydroxyaspergillumarin A (**96**) were new, and the absolute configurations of **95**, **96**, **98**, and **99** were confirmed via a modified Mosher’s ester method. All compounds were demonstrated to be inactive against a group of bacteria and fungi, including *Staphylococcus aureus*, *Escherichia coli*, *Mycolicibacterium smegmatis*, *Candida albicans*, and *Aspergillus niger*.

Four new diphenylpyranone compounds, dihydroaltenuene A (**101**) and B (**102**), and de hydroal tenu ene A (**103**) and B (**104**), and three known compounds **105**–**107** (Figure 8), were isolated from an unidentified aquatic fungal isolate A–00471, inhabited in the Cheoah River in the Great Smoky Mountains, Tapoco, North Carolina [35]. In antibiotic assays for *Bacillus subtilis* (ATCC 6051), *Staphylococcus aureus* (ATCC 29213), and *Escherichia coli* (ATCC 25922), compounds **101**, **103**, **104**, isoaltenuene (**105**), and altenuene (**106**) were active toward *B. subtilis*, causing zones of inhibition of 50, 13, 20, 30, and 20 mm at 100 µg/disk, respectively. Compounds **101**, **104**, and **106** showed activity against *S. aureus*, affording 14, 14, and 18 mm zones of inhibition at the same level, respectively. All compounds were inactive against *E. coli* at this level. Interestingly, both compound **102** and 5′–epialtenuene (**107**) did not display activity in these antibacterial assays, despite differing from **101** and **106** only in the relative stereochemistry at C–5′ (Figure 8). Orfali et al. cultivated the thermophilic fungus *Penicillium* sp. RO–11 from Ghamiqa hot spring sediments in the south of Saudi Arabia, and obtained a new pyran analog, named 3–(furan 12–carboxylic acid)–6–(methoxycarbonyl)–4–hydroxy–4–methyl–4, 5–dihydro–2*H*–pyran (**108**), and a known austinol (**109**) (Figure 8) [14]. It is noteworthy that compound **108** displayed potent activity against *Pseudomonas aeruginosa* (NR117678.1) and *Escherichia fergusonii* (CU928158.2) with respective MIC value of 7.4 ± 2.4 and 9.3 ± 10.2 µg/mL, and compound **109** showed a broad–spectrum and highly effective antibacterial activity against *P.aeruginosa*, *Staphylococcus aureus* (CP011526.1) and *E. fergusonii* with MIC values of 0.13 ± 0.4, 1.4 ± 2.4, and 2.5 ± 1.7 µg/mL, respectively. Moreover, compound **109** also exhibited antiproliferation function to the lymphoma human cancer cell line HTB–176 with an IC_50_ value of 10 ± 3.92 μM in the MTT assay, whereas compound **108** was inactive against these tumor cells.

### 2.4. Benzoquinone, Phenol, Phenolic Acid and Their Derivatives

Screening and isolation of natural products with antibacterial activity of freshwater fungus *Camposporium quercicola* YMF1.01300 from Yunnan province of China resulted in the discovery of compound 2′,4′–dihydroxyacetophenone (**110**) (Figure 9) [36]. This compound showed antagonistic activity against *Bacillus cereus* (YMF 3.19), *B. laterosporus* (YMF 3.08), and *Staphylococcus aureus* (YMF 3.17) at the concentration of 200 µg/disk, but no activity against *Escherichia coli* (YMF 3.16). Besides eight new annularins A–H (**23**, **24**, and **59**–**64**) (Figure 2 and Figure 5), the fungus *Annulatascus triseptatus* A–353–1B also biosynthesized (−)–(*S*)–*p*–hydroxyphenyllactic acid (**111**) (Figure 9), whose bioactivity was not mentioned [24]. A new phenolic compound with the distinguished aliphatic side–chains present and unusual ethyl group, named anguillospral (**112**) (Figure 9), was isolated from the freshwater fungus *Anguillospora longissima* CS–869–1A, which was associated with birchwood baits placed in Jordan Creek, a tributary of the Salt Fork River, Vermillion County, Illinois, USA [37]. This compound showed antagonistic effects toward *Staphylococcus aureus* (ATCC 29213) and *Candida albicans* (ATCC 90029) with MIC values of 4 and 58 µg/mL, respectively. Besides the furanone and pyrone metabolites (compounds **25**–**28** and **45** and **46** as shown in Figure 2 and Figure 5, respectively) as stated earlier, a known benzoic acid (**113**) (Figure 9) was obtained from the freshwater–derived fungus *Massarina tunicate* (A–25–1). With the exception of *Staphylococcus aureus* ATCC 29213, *Candida albicans* ATCC 14053, *Aspergillus flavus* NRRL 6541, and *Fusarium verticillioides* NRRL 25457, this compound was only able to inhibit the growth of *Bacillus subtilis* (ATCC 6051) at 200 µg/disk, causing a zone of inhibition of 15 mm [25]. From the fungus *Penicillium* sp. RO–11, a new 3*α*–methyl–7–hydroxy–5–carboxylic acid methyl ester–1–indanone (**114**) (Figure 9), along with the two mentioned above pyran compounds **108** and **109** (Figure 8), were obtained by Orfali et al. [14]. The new compound **114** was active toward the pathogenic bacteria *Pseudomonas aeruginosa* and *Escherichia fergusonii* with MIC respective value of 5.4 ± 5.9 and 6.3 ± 7.8 µg/mL, and was active against human lymphoma cells HTB–176 with an IC_50_ value of 22 ± 2.94 μM. The aquatic fungus *Ophioceras dolichostomum* YMF1.00988 was isolated from a submerged woody substrate collected from a freshwater habitat in Yunnan Province, China [38]. The screening for biologically active metabolites of this strain, YMF1.00988, resulted in two known compounds, **115** and **116**, and a novel fungal metabolite, neolignan ophiocerol (**117**), with dibenzo–1,6–dioxacyclodecane carbon skeleton (Figure 9). All three compounds were evaluated for antifungal activity against eight plant pathogens, including *Exserohilum turcicum* YMF 1.1990, *Fusarium* sp. YMF 1.1996, *Paecilomyces lilacinus* YMF 1.621, *Phyllosticta* sp. YMF 1.1995, *Alternaria* sp. YMF 1.1997, *Aspergillus niger* YMF 1.46, *Coleosporium* sp. YMF 1.2088 and *Colletotrichum* sp. YMF 1.2099. These compounds all showed similar moderate antifungal activity. Specifically, compound caffeic acid (**115**) afforded the inhibition zones ranging from 7 to 17 mm at 50 µg/disk, isoamericanoic acid A (**116**) and compound **117** produced the respective inhibition zones of 7–11 mm and 6–11 mm at the same level. Moreover, the nematicidal activity of these three compounds against *Bursaphelenchus xylophilus* was also evaluated. Both compounds **115** and **116** showed potency with LC_50_ values of 46.8 and 133.7 mg/mL, while compound **117** was not active. The fungus *Lindgomyces madisonensis* G416 was isolated from submerged wood collected in a stream in North Carolina, USA. It was fermented on rice solid–substrate culture and extracted by CHCl_3_–CH_3_OH (v:v 1:1), yielding seven acetophenone derivatives **118**–**124** (Figure 9). Among them, compounds madisone (**118**), 4′–methoxymadisone (**119**), and dihydroallovisnaginone (**122**) displayed antimicrobial activity against *Staphylococcus aureus*, *Escherichia coli*, *Mycobacterium smegmatis*, *Candida albicans*, and *Aspergillus niger* with MIC values greater than 55 µg/mL [39].

Four new diphenyl ethers, named tenellic acids A–D (**125**–**128**) (Figure 10), were obtained from the aquatic fungus *Dendrospora tenella* CCM F–10787 from foam collected from Allen Creek (a freshwater stream) in Wood Point, New Brunswick, Canada [40]. All of them were evaluated for antagonistic activity against Gram–positive bacteria through standard disk assays at 200 µg/disk and found to prevent the growth of the bacterial strain *Bacillus subtilis* (ATCC 6051), affording zones of inhibition of 11, 9, 12, and 29 mm, respectively. Compounds **127** and **128** were also active toward *Staphylococcus aureus* (ATCC 29213), causing inhibitory zones of 14 and 25 mm, respectively. However, none of them displayed activity against *Candida albicans* (ATCC 14053). The fungus *Phoma* sp. TPU1222 was isolated from a freshwater sample in Hayakake Lake, Aomori, Japan [41]. Bioassay–guided separation of the EtOAc extract of the strain TPU 1222 fermentation broth yielded a new biphenyl ether derivative, 1–methoxy–3,5′–dimethyl–2,3′–oxybiphenyl–5,1′,2′–triol (**129**), together with two known phenolic compounds, 5–methoxy–3,5′–dimethyl–2,3′–oxybiphenyl–1,1′,2′–triol (**130**) and cyperine (**131**) (Figure 10). The inhibitory effects of these three compounds on protein tyrosine phosphatase 1B (PTP1B), T–cell protein tyrosine phosphatase (TCPTP), CD45 tyrosine phosphatase (CD45), and Vaccinia H–1–related phosphatase (VHR) were evaluated. Both compounds **129** and **131** exhibited inhibition for PTP1B with respective IC_50_ values of 13 and 17 μM, but compound **130** only showed 32% inhibition at 36 μM. Meanwhile, compounds **129** and **131** showed similar inhibitory activity against VHR, weaker inhibitory activity against TCPTP and CD45, and IC_50_ values of 36 μM against PTP1B. In addition, compound **131** exhibited modest cytotoxicity (26% growth inhibition at 50 μM), whereas compounds **129** and **130** did not affect cell proliferation at the same level. Wang et al. [42] obtained a new diphenyl ether quercilolin (**132**) and a known tenellic acid A (**125**) (Figure 10) that was also isolated from the freshwater fungus *D. tenella* CCM F–10787 mentioned above, from the fungus *Camposporium quercicola* YMF 1.01300 associated with decaying woods submerged in lakes in Yunnan Province, China. Both compounds had antagonistic activity versus *Bacillus cereus* YMF 3.19, *B. laterosporus* YMF 3.08, and *Staphylococcus aureus* YMF 3.17 at 200 µg/disk, with compound **132** producing 16, 15, and 19 mm inhibition zones and compound **125** affording 18, 14, 16 mm inhibition zones, respectively. However, neither of them was effective against *Escherichia coli* YMF 3.16. Two new chlorinated diphenyl ethers, 2,4–dichloro–3–(2–chloro–3–hydroxy–5–methylphenoxy)–5–methylphenol (**133**) and 3,3′–oxybis(2,4–dichloro–5–methylphenol) (**134**) (Figure 10), were obtained from the fungal strain C–76–1(ATCC 76730) belonging to an undescribed species of *Kirsrhsteiniotbelia*, which was isolated from submerged wood in a natural thermal stream in Puyehue, Chile [42]. In disk assays, these two new compounds showed antibacterial activity against *Bacillus subtilis* and *Staphylococcus aureus* at 5 and 1 µg/disk, respectively.

The secondary metabolites of the freshwater aquatic fungus *Wicklowia aquatic* (F76–1) have good chemical diversity, comprising the mentioned nonadride derivatives **11**–**16** and waquafranones **17** and **18** (Figure 1), three benzoic acid analogs **135**–**137** (Figure 11), and one known compound folipastatin (**138**) (Figure 11) [20]. The bioactivity of compounds **135**–**138** was not described in Hosoe et al. [20]. The freshwater fungus *Helotiales* sp. (G730) occurring on submerged wood in a freshwater lake in Hanging Rock State Park, North Carolina, UAS, could produce three new prenylated diresorcinols, leotiomycenes A–C (**139**–**141**) (Figure 11) [43]. The structure of compound **139** was determined via X–ray diffraction, and the absolute configuration of the new compound was determined by TDDFT–ECD and optical rotation calculation. What is noteworthy is that all three new compounds could suppress quorum sensing by inhibiting autoinducing peptides (AIP) production in methicillin–resistant *Staphylococcus aureus* (MRSA) (AH1263) with IC_50_ values ranging from 0.3 to 12.5 μM, and leotiomycene A (**139**) was considered as a lead compound of AgrA inhibitor because of its highly inhibitory advantage over the positive control ambuic acid.

### 2.5. Naphthalene and Naphthalenone Derivatives

Four known naphthalenones **142**–**145** (Figure 12) were obtained from the freshwater fungus *Caryospora callicarpa* YMF1.01026 isolated from a submerged woody substrate in Yunnan Province, China [44,45]. All three compounds exhibited killing activity against the nematode *Bursaphelenchus xylophilus* with LC_50_ values of 206.1, 103.1, 105.8, and 105.1 ppm, at 36 hr, respectively. It is worth noting that compound 3,4,6,8– tetrahydroxy–3,4–dihydronaphthalen–1(2*H*)–one (*cis*–4–hydroxyscytalone) (**145**), replaced by 3–OH, is more active than the other similar compounds **142**–**145**. The fungus *Paraphoma radicina* G104 from a freshwater habitat was also found to produce isosclerone (**146**) and radinaphthalenone (**147**) (Figure 12), besides a furanone analog (**29**) (Figure 2) and six pyranones **88**–**93** (Figure 7) [26]. Both naphthalenone derivatives **146** and **147** showed no activity against the bacteria of *Staphylococcus aureus*, *Escherichia coli*, and *Mycobacterium smegmatis*, and the fungi of *Saccharomyces cerevisiae* and *Aspergillus niger* at a concentration of 63 μg/mL. The fungus *Delitschia* sp. G858 was able to biosynthesize not only α–pyrone compounds **47**–**58** (Figure 5) but also two naphthalenones **148** and **149** (Figure 12). Compound **148** was identified as the new 3*S*∗,4*S*∗–7–ethyl–4,8–dihydroxy–3,6–dimethoxy–3,4–dihydronaphthalen–1(2*H*)–one. Both compounds **147** and **148** had cytotoxicity against the African American prostate cancer cell line (E006AA–hT) at 40 µM [32]. The pyran metabolite (**44**) (Figure 4) mentioned above and four naphthalenones, including a new (3R*,4S*)–7–ethyl–3,4,6,8–tetrahydroxy–3,4–dihydronaphthalen–1(2*H*)–one (**150**) and three known compounds **151**–**153** (Figure 12), were obtained from the freshwater–derived *Delitschia corticola* YMF 1.01111 [12]. At 50 μg/disk, compounds **150**–**153** were all active against the previously mentioned six microbial pathogens. To be more specific, these four compounds exhibited moderate antifungal activity against *Alternaria* sp. YMF 1.01991, *Sclerotium* sp. YMF 1.01993, and *Fusarium* sp. YMF 1.01996 with zones of inhibition ranging from 7 to 9 mm, and displayed strong antibacterial activity against *Bacillus cereus* YMF 3.19, *B. laterosporus* YMF 3.08, and *Staphylococcus aureus* YMF 3.17 with zones of inhibition ranging from 10 to 20 mm.

The first study on natural products of the member belonging to the fungal genus of *Astrophaeriella* was conducted by Wang et al. [46]. Three new naphthoquinone pigments, astropaquinones A–C (**154**–**156**) (Figure 13), were obtained from the fungus *A. papuana* YMF 1.0118, which was isolated from a woody substrate submerged in freshwater in Yunnan Province, China. Astropaquinones B (**155**) and C (**156**) were found to possess a rare pyranonaphthoquinone skeleton containing a lactol ring (Figure 13). All three naphthoquinone compounds **154**–**156** were active toward the two fungal strains, *Alternaria* sp. YMF 1.01991 and *Alternaria* sp. YMF 1.01997 with inhibitory zones ranging from 7 to 13 mm, and three bacterial strains of *Bacillus cereus* YMF 3.19, *B. laterosporus* YMF 3.08, and *Staphylococcus aureus* YMF 3.17 with inhibitory zones ranging from 9 to 20 mm. Astropaquinone B (**155**) also showed activity against *Escherichia coli* YMF 3.16, *Gibberella saubinetii* YMF 1.01989, *Phyllosticta* sp. YMF1.01995, and *Fusarium* sp. YMF 1.01996, causing zones of inhibition of 10, 11,16, and 12 mm, respectively, while astropaquinone B (**156**) also displayed activity against the above three fungal pathogens and the fungus *Colletotrichum* sp. YMF 1.01994, affording zones of inhibition of 9, 13, 9, and 9 mm, respectively. The results of antimicrobial activity indicated that the presence of the pyran ring may be an important functional group in this type of naphthoquinone (Figure 13). The fungal strain *Minutisphaera parafimbriatispora* G156–4 was isolated from a submerged wood in a swamp area of the USA (North Carolina) [47]. After 28 days of rice fermentation incubation, two known aromatic polyketides, isosclerone (**157**) and sphaerolone (**158**) (Figure 13), were obtained from the MeOH/CHCl_3_ (*v*:*v* 1:1) extraction of fermentation products. Compound **158** showed antibacterial activity against *Staphylococcus aureus* and *Mycobacterium smegmatis* with MIC values of 30 and 60 µg/mL. Furthermore, compound **157** was also obtained from another freshwater–derived fungus, *M. aspera* (G427), derived from the same woody sample. The chemical investigation of the liquid fermentation of the fungus *Kirschsteiniothelia funicularia* (C–76–1), associated with submerged wood collected from a natural thermal stream in Puyehue, Chile, led to the isolation and identification of seven naphthoquinone compounds **159**–**165** (Figure 13) [42]. Kirschsteinin (**164**) was then first reported as a rare example of naphthoquinone dimer with the monomer units connected by an ethylidene linkage. Compound **164** had the ED_50_ values against three human solid tumor cell lines, including human non–small lung carcinoma A–549, breast adenocarcinoma MCF–7, and colon adenocarcinoma HT–29 cells, which were 3.1, 5.2, and 2.7 µg/mL, respectively. This compound also showed antagonistic activity against *Bacillus subtilis* and *Staphylococcus aureus* at 1.0 and 10 µg/disk, respectively.

It was demonstrated that the freshwater sediment–derived fungus *Penicillium* sp. (S1a1) could produce at least three types of secondary metabolites, including isochromans **41**–**43**, as previously shown in Figure 4, three new tanzawaic acid derivatives penitanzchroman (**166**), tanzawaic acids Y (**167**) and Z (**168**), and six known compounds **169**–**174** (Figure 14), and anserinones A (**306**) and B (**307**) to be mentioned below [31]. Among these nine tanzawaic acids **166**–**174**, only tanzawaic acid B (**171**) exhibited weak antibacterial activity against *Staphylococcus aureus* (ATCC 700699) with an MIC value of 50 µM, and all compounds were inactive in the cytotoxicity assay for the mouse lymphoma cell line (L5178Y).

Besides three pyran derivatives **36**–**38** (Figure 3) [29,48], Dong et al. [29,48,49] also obtained twelve naphthalenes and naphthalenons from the unidentified fungus YMF 1.01029, including two known naphthalenone metabolites **175** and **176** [49], two novel naphthalene–containing compounds colelomycerones A (**177**) and B (**178**) [48], five new preussomerin analogs ymf 1029A–E (**179**–**183**) [49], and three known preussomerin C (**184**) [49], preussomerin D (**185**) [48,49], preussomerin E (**186**) (Figure 15) [29]. Two known naphthalenones, **175** and **176**, and seven preussomerins, **179**–**185**, exhibited nematicidal activity against nematode *Bursaphelenchus xylophilus* with IC_50_ values ranging from 200 to 100 μg/mL, among which compound **185** was the most potent, and compounds **175** and **176** were the weakest ones. Moreover, compounds **177**, **178**, **185**, and **186** displayed antimicrobial activity against the bacteria *Bacillus subtilis* YMF 3.19, *B. laterosporus* YMF 3.08, and *Staphylococcus aureus* YMF 3.17 and the fungi *Bipolaris maydis* YMF 1.2094, *Cochliobolus sativus* YMF 1.2088, and *Fusarium verticillioides* YMF 1.2076, with 11–20 mm inhibition area at 50 µg/disk, while compound **185** exhibited the most noticeable activity. The analysis of SAR suggested that the bis–spirobisnaphthalene/naphthalenone carbon skeleton should be critical for making nematocides with greater efficiencies.

Jiao et al. [50] obtained six dimeric naphthalene spiroketal–type compounds, decaspirones A–E (**187**–**191**) and palmarumycin CP1 (**192**) (Figure 16), from the aquatic fungus *Decaisnella thyridioides* A–00267–2A isolated from submerged, decorticated wood in the Lemonweir River in Wisconsin, with **187**–**191** being the new compounds. The structure of decaspirone A (**187**) was assigned using X–ray crystallographic analysis, and its absolute configuration was determined utilizing the modified Mosher method. Structurally, the naphthalene ring system of compounds **187**–**191** is *trans*–coupled, whereas the naphthalene ring system of all previously discovered compounds of this type is *cis*–coupled. In the antimicrobial assays for new compounds **187**–**191**, all the five compounds showed activity against *Bacillus subtilis* (ATCC 6051) with the respective zone of inhibition of 39, 19, 34, 30, and 30 mm at 50 µg/disk, and compounds **187**, **188**, and **191** exhibited activity against *Staphylococcus aureus* (ATCC 29213) with the diameters of inhibitory area were 41, 30, and 28 mm at 100 µg/disk, respectively. Meanwhile, compounds **187**, **188**, and **189** displayed antagonistic effects on the growth of the fungal strain *Candida albicans* ATCC 14053, causing inhibition zones of 30, 13, and 17 mm at 100 µg/disk, respectively. In addition, compound **187** had antifungal activity against *Aspergillus flavus* NRRL 6541 and *Fusarium verticillioides* NRRL 25457 with MIC values of 10 and 5 μg/mL, while compound **191** was less active with an MIC value of 25 µg/mL against *F. verticillioides* but was not active against *A. flavus* at the same concentration.

### 2.6. Anthraquinone and Xanthone Derivatives

A known emodin (**193**) (Figure 17) was isolated from the thermophilic fungus *Penicillium* sp. RO–11, which was previously mentioned as the fungal strain producing compounds **108** and **109** (Figure 8) and **114** (Figure 9) [14]. This compound displayed antibacterial activity against *Pseudomonas aeruginosa* with an MIC value of 12.5 ± 14.2 µg/mL, and cytotoxicity against HTB–176 human lymphoma cells with an IC_50_ value of 2 ± 7.6 μM. A known anthraquinone compound **194** (Figure 17) and the mentioned three new naphthoquinone pigments astropaquinones A–C (**154**–**156**) (Figure 13) mentioned above were obtained from the fungus *Astrophaeriella papuana* YMF 1.01181 [46]. Compound **194** showed moderate antimicrobial activity against *Alternaria* sp. YMF 1.01991, *Alternaria* sp. YMF 1.01997, *Bacillus cereus* YMF 3.19, *B. laterosporus* YMF 3.08, and *Staphylococcus aureus* YMF 3.17, with inhibitory zones ranging from 8 to 11 mm. Three known compounds **195**–**197** (Figure 17) were obtained from the fungus *Chaetomium* sp. YMF 1.02105 is associated with submerged wood in the River Bailong in Kunming, Yunnan Province, China [51]. The bioassay of these three compounds was not conducted at that time. Four new trihydroxyxanthone derivatives, xanthones I–IV (**198**–**201**) (Figure 17), along with the aforementioned mansonone D (**22**) (Figure 2) was isolated from the aquatic fungus *Chaetosphaeriaceae* sp. BCC 28210 [22]. All the isolated compounds were evaluated for antimalarial, antimicrobial, and cytotoxic activity. Among them, compound **199** was active against *Plasmodium falciparum* K1, *Candida albicans*, and Vero (African green monkey kidney fibroblast) cells with respective IC_50_ values of 7.75, 18.4, and 19.1 µg/mL, and compound **201** was active against *Bacillus cereus* with an MIC value of 6.25 µg/mL and Vero cell lines with an IC_50_ value of 47.9 µg/mL.

The freshwater fungi *Clohesyomyces* sp. G102 was found to produce three new xanthone derivatives, including a monomeric tetrahydroxanthone (**202**) and a pair of monomeric hexahydroxanthones **203** and **204**, and a known homodimeric tetrahydroxanthone (**205**) (Figure 18) besides α–pyrone compounds **65**–**76** (Figure 6) [33]. The relative configurations of compounds 8–hydroxyblennolide H (**202**), *cis*–dihydro–8–hydroxyblennolide H (**203**), and *trans*–dihydro–8–hydroxyblennolide H (**204**) were determined based on their NOE data. Compounds **202**, **203**, and **205** were found to inhibit the essential enzyme peptidyl–tRNA hydrolase (Pth1) of *Salmonella typhimurium*, with secalonic acid A (**205**) being the most potent (IC_50_ = 1.4 mM). However, compounds **202**–**205** have no antimicrobial activity against a panel of pathogenic microbes mentioned above. A new antibiotic, called quinaphthin (**206**) (Figure 18), was obtained from the fungus *Helicoon richonis* hosted in submerged wood collected from Bystock Reservoir, Devon, UK [10]. The structure of compound **206** was determined by X–ray crystallographic analysis. Quinaphthin (**206**) exhibited a broad–spectrum antagonistic activity against a series of pathogens, including three bacteria of *Staphylococcus aureus*, *β–haemolytic Streptococcus*, and *Bacillus cereus*, three fungi of *Candida albicans*, *Saccharomyces cerevisiae*, and *Aspergillus niger*, two wall–less bacteria of *Acholeplasma laidlawii* and *Mycoplasma gallisepticum*, and the human pathogen *Trichomonas vaginalis*), comparable to the positive drug metronidazole. However, this compound is more toxic than the anticancer agent doxorubicin, which makes its utility impossible as antibiotics.

The fungal strain *Aspergillus* sp. TPU1343 was isolated from a freshwater sample in Okinawa, Japan [52]. The separation of the EtOAc extract of strain TPU1343 fermentation broth yielded an unusual dimer of tetrahydroxanthone through an ether bond, named asperdichrome (**207**), and two known dimeric polyketides, secalonic acids D (**208**) and F (**209**) (Figure 19). These three compounds were tested for the inhibitory effects on the activity of protein tyrosine phosphatase (PTP) 1B that plays a key negative role in insulin signaling pathways of type 2 diabetes. Compounds **207** and **209** could inhibit PTP1B with respective IC_50_ values of 6.0 and 9.6 μM, but compound **208** showed only 40% inhibition at 15.7 μM. Therefore, the heterodimeric structures presented in compounds **207** and **209** appear to be more favorable for enzyme inhibition. Next year, two compounds, secalonic acids F (**210**) and F1 (**211**) (Figure 19), were also isolated from the same fungus strain TPU1343 [53]. As shown in Figure 19, the structure of compound **210** possesses a 2,2′–linked bis–tetrahydroxanthone skeleton, which is ubiquitous in the members of the secalonic acid family, while compound **211** features a heterodimeric tetrahydroxanthone skeleton, which is similar to the previously obtained compounds **207**–**209**. In addition, compound **211** had IC_50_ values of 5.9, 6.9, 14, and 6.2 μM against PTP1B, T–cell PTP (TCPTP), CD45 tyrosine phosphatase (CD45), and Vaccinia H–1–related phosphatase (VHR), respectively.

### 2.7. Depsidones

Besides three known anthraquinone and xanthone derivatives **195**–**197** as shown in Figure 17, six new dibenzo [*b*,*e*] oxepinone metabolites, chaetones A–F (**212–217**) (Figure 20) were obtained from the fungus *Chaetomium* sp. YMF 1.02105 [51]. All six new compounds displayed various degrees of inhibitory activity against five tumor cell lines of A549, Raji, HepG2, MCF–7, and HL–60. Among them, chaetone C (**214**) showed the most potent cytotoxicity against all the above tumor cells with IC_50_ values of 1.2, 1.8, 1.9, 2.3, and 1.6 µg/mL, respectively, which was while chaetone F (**217**) exhibited moderate antiproliferative effects on these five cell lines with IC_50_ values of 8.1, 7.8, 6.7, 5.9, and 9.1 µg/mL, respectively. The analysis of SAR suggested that the hydrophobicity of the dibenzo [*b*,*e*] oxepin core in compound **214** may be of great significance to the cytotoxic activity of arugosin–type dibenzophenones. In addition, chaetones B–F (**213**–**217**) showed moderate antibacterial activity against *Staphylococcus aureus* (ATCC 6538) at 50 μg/disk, with the inhibition zones size of 11–15 mm, but no antifungal activity against *Aspergillus fumigatus* (ATCC 10894), *Candida albicans* (ATCC 10231), and *Geotrichum candidum* (AS2.498) at the same concentration. It was reported that the chemical skeleton of dibenzo [*b*,*e*] oxepinones (as shown in Figure 20) was rare in the dibenzophenone class of compounds. Pestalone (Figure 20), as the most remarkable dibenzo[*b*,*e*]oxepinone with antibiotic properties, whose structure is close to that of these natural products, could be of significant biomedical importance.

### 2.8. Macrolides

The first report of secondary metabolites from the fungal genus *Caryospora* is that three novel tetradecalactones, caryospomycins A–C (**218–220**) (Figure 21), were isolated from the aquatic fungus *Caryospora callicarpa* YMF1.01026 of a submerged woody substrate in Yunnan Province, China [44,45]. The fungal strain YMF1.01026 also produces naphthalenones **142**–**145** (Figure 12), as mentioned above. The chemical structures of caryospomycins were determined to belong to the 14–membered macrolides featuring a fused 1,2–dimethoxy–4–hydroxybenzene ring (Figure 21), which is rare in the natural resorcylides. These compounds were active in killing the nematode *Bursaphelenchus xylophilus* with the LC_50_ values of 207, 229.6, and 220.3 ppm, respectively, at 36 h. As shown in Figure 21, fourteen new resorcylic acid lactones (**221**–**234**), including greensporones A–G (**221**–**227**), 8,9–dihydrogreensporones A, C, and D (**228**–**230**), dechlorogreensporones A (**231**), D (**232**) and F (**234**), and *O*–desmethylgreensporone C (**233**), were isolated from the organic extract of the freshwater fungus *Halenospora* sp. G87 occurring on a wood substrate submerged in a stream in North Carolina, USA [54]. The absolute configuration of compound 8,9–dihydrogreensporones C (**229**), selected as a representative member, was determined via X–ray diffraction analysis, and compounds greensporones D and E (**224** and **225)**, 8,9–dihydrogreensporones D (**230**), and dechlorogreensporones D (**232**) were determined using a modified Mosher’s ester approach. The cytotoxic for the MDA–MB–435 (melanoma) and HT–29 (colon) cancer cell lines was evaluated. The compound greensporone C (GC, **223**) was the most potent with IC_50_ values of 2.9 and 7.5 µM, respectively. The analysis of SAR of compounds **221**–**223**, **225**, and **227**–**234** suggested that the presence of the (*E*)–enone should be critical for cytotoxicity, the chelated phenolic functionality of position C–16 should reduce cytotoxicity of greensporone C (**223**), and the hydroxylation of position C–5 in compound **232** and oxidation of the C–5 to the ketone in compounds **221** and **231** should diminish the cytotoxicity of these compounds for MDA–MB–435 cells. Afterwards, the study on the apoptotic mechanism of K562, U937, and AR320 leukemia cells, greensporone A (GA, **221**) and GC (**223**) was conducted by Prabhu et al. [55,56]. Treatment with GC (**223**) led to down–regulation of apoptosis protein inhibitors (XIAP, cIAP–1, cIAP–2) and up–regulation of pro–apoptotic proteins (Bax). The up–regulation of Bax is associated with the release of cytochrome c. Released cytochrome c further activates the caspase cascade and initiates the apoptosis process. However, treatment with GA (**221**) resulted in down–regulation of the anti–apoptotic genes IAP and Bcl–2. The most obvious difference between these two compounds is the carbonylation of C–5 and the chlorination of C–13 (Figure 21), which could generate steric hindrance and might negatively affect binding and reduce the lipophilicity of greensporones, thus weakening the cytotoxicity of GA (**221**) against leukemia cells.

### 2.9. Polyesters

**Figure 22:**
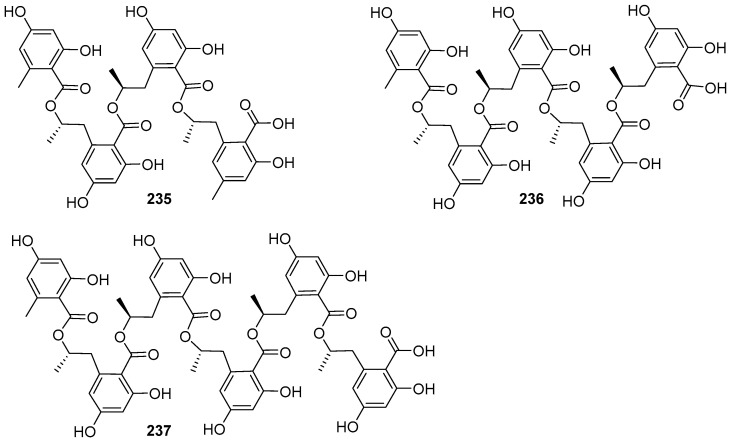
Chemical structures of compounds **235**–**237** from *Ascomycete* sp.

Three new linear polyester compounds W1278–A––C (**235**–**237**) (Figure 22) were obtained from the aquatic fungus *Ascomycete* sp. LL–W1278, which was isolated from a freshwater sample in Tai Po Kau nature reserve, Hong Kong, China [57]. However, their biological activities were not mentioned in Schlingmann et al. [57].

### 2.10. Alkaloids

**Figure 23:**
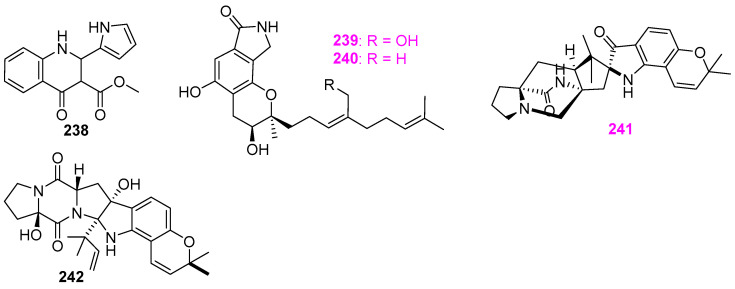
Chemical structures of compounds of **238** from *Penicillium* sp., **239** and **240** from *Stachybotrys* sp., and **241** and **242** from *Aspergillus ochraceus*.

From the fungus *Penicillium* sp. RO–11 living in hot spring, 2–methyl–penicinoline (**238**) (Figure 23), together with pyran and pyranone compounds **108** and **109** (Figure 8), indanone analog **114** (Figure 9), and a known anthraquinone derivative (**193**) (Figure 17), as stated earlier, were obtained [14]. Compound **238** was inactive toward the aforementioned five pathogens at 25 µg/mL and HTB–176 human lymphoma cells. Two new aromatic alkaloids, stachybotrins A (**239**) and B (**240**) (Figure 23), were obtained from the liquid fermentation cultures of the fungus *Stachybotrys* sp. (CS–710–1) isolated from brackish water in Florida, USA [58]. The pyrano and isodihydroindole chemical skeleton in the stachybotrins was first reported as a characteristic structure presented in natural products at that time. Both compounds displayed antibacterial activity against *Bacillus subtilis* (ATCC 6051) at 10 µg/disk, and also showed antifungal activity against *Ascobolus furfuraceus* (NRRL6460) and *Sorduria fimicola* (NRRL6459) at 10 and 20 µg/disk, respectively. In addition, compound **239** exhibited mild antiproliferation activity against three human tumor cell lines nonsmall carcinoma A–549, breast adenocarcinoma MCF–7, and colon adenocarcinoma HT–29, with ED_50_ values ranging from 20 to 30 µg/mL. In the search for novel compounds with bioactivity, the fungus *Aspergillus ochraceus* KM007 in the freshwater of Fuxian Lake in Yunnan province of China was found to produce two new prenylated indole alkaloids speramides A (**241**) and B (**242**) (Figure 23), among which compound **242** was the first discovery of a natural prenylated brevianamide derivative at the time [15]. Both compounds were tested for antibacterial activity against methicillin–resistant *Staphylococcus aureus* (MRSA) 92^#^, MRSA 98^#^, *S. aureus*, and *Pseudomonas aeruginosa*, and cytotoxicity against PC3, DU145, and Lncap cell lines. However, only compound **241** was active toward *P. aeruginosa* with an MIC value of 0.8 μM.

**Figure 24:**
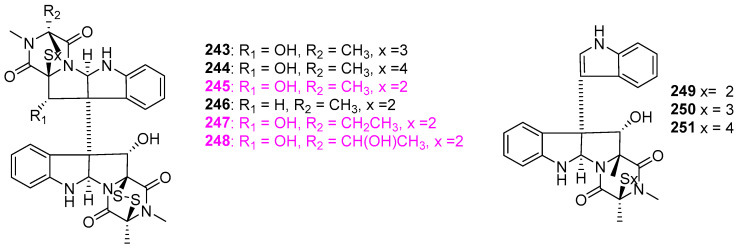
Chemical structures of compounds **243**–**251** from *Gliocladium roseum*.

Five new gliocladines A–E (**243**–**247**) and four known **248**–**251** (Figure 24) were isolated from the methanol extract of wheat solid–substrate fermentation of *Gliocladium roseum* 1A, which was isolated from submerged wood in freshwater in Yunnan Province, China [59]. The nematicidal assay indicated that both *Caenorhabditis elegans* and *Panagrellus redivivus* were sensitive to all nine compounds, and compound **249** was the most potent with an ED_50_ value of 10 μg/mL. However, no antinematodal effect of these nine compounds on *Bursaphelenchus xylophilus* was observed at concentrations up to 400 μg/mL. Compared to the compounds **243**, **244**, and **248**–**251**, the monomeric epipolysulfanydioxopiperazines (**245**–**247**) (Figure 24) with the indole moiety were less active, which suggested that the number of sulfur atoms in the dioxopiperazine rings should have no influence on the nematicidal activity of this class of compounds. Moreover, compounds **245**, **247**, and **248**, named as verticillin A, sch52901, and sch52900, were also obtained from *Gliocladium* sp. SCF–1168 from freshly fallen dicot leaf litter in the El Yunque rain forest of Puerto Rico [60]. The mouse BALB/c clone A31 cells that carried the *fos/lac* Z reporter gene but lacked the cyclic AM response element were employed in the evaluation for antineoplastic activity. These three compounds exhibited an inhibitory activity in vitro with IC_50_ respective value at 1.5, 18, and 0.5 μM. Verticillin A (**245**), as the most potent inhibitor for BALB/c 3T3 cells, was chosen for the mechanistic studies of antitumor activity. This compound could be acting at a very early step in the signalling pathway, which is responsible for activation of multiple signalling pathways leading to *c–fos* proto–oncogene induction.

### 2.11. Peptides

**Figure 25:**
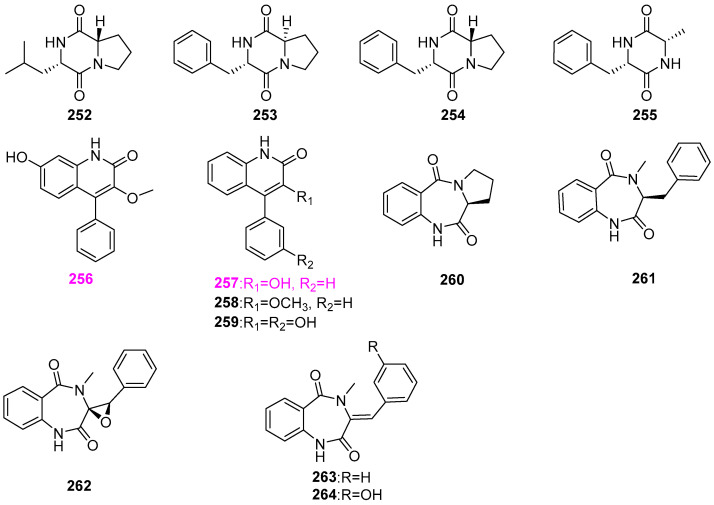
Chemical structures of compounds of **252**–**255** from *Minutisphaera aspera*, and **256**–**264** from *Myrothecium verrucaria*.

The chemical investigation of the fungus *Minutisphaera aspera* (G427), producing naphthalenone compound **157** (Figure 13), stated earlier, yielded four known dipeptides **252**–**255** (Figure 25) [47]. However, all of the four dipeptides were inactive against *Staphylococcus aureus*, *Escherichia coli*, *Mycobacterium pubescens*, *Candida albicans*, and *Aspergillus niger* at 60 μg/mL. One new quinolinone, 7–hydroxy–3–methoxyviridicatin (**256**), as well as eight known compounds **257**–**264** (Figure 25), were obtained from the freshwater–derived fungus *Myrothecium verrucaria*, which was isolated from the lake of Chenghai in Yunnan Province of China [61]. Among them, the new compound **256** showed antibacterial activity against all the tested pathogenic bacteria at 25 μM, including *Bacillus cereus*, *B. subtilis*, *Vibrio anguillarum*, and *V. parahaemolyticus*, and compound **257** was also active toward the above three pathogens, with the exception of *B. subtilis*, at the same level.

**Figure 26:**
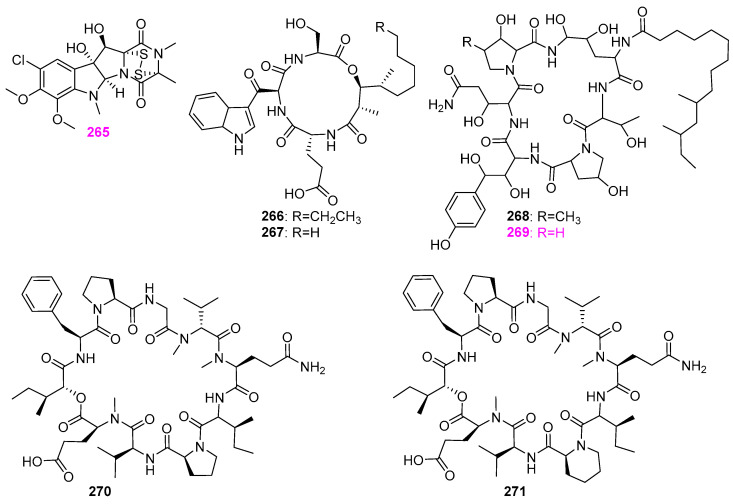
Chemical structures of compounds **265**–**267** from *Delitschia* sp., **268** and **269** from *Glarea lozoyensis*, and **270** and **271** from *Clohesyomyces aquaticus*.

The secondary metabolites of the fungus *Delitschia* sp. G858 had good chemical diversity. It had been demonstrated that this fungal strain could produce the mentioned α–pyrone compounds **47**–**58** (Figure 5) and two naphthalenones **148** and **149** (Figure 12), and three peptides: sporidesmin A (**265**), artrichitin (**266**), and lipopeptide 15G256ε (**267**) (Figure 26) [32]. Of these three peptides, only compound **265** displayed potent inhibition for the viability of the African American prostate cancer cell line (E006AA–hT) at 2.5 µM. This compound was also found to be derived from another aquatic fungus, *Delitschia corticola* YMF 1.01111 mentioned above, and exhibited a broad–spectrum antimicrobial activity against a panel of bacteria and fungi, as Sun et al. [12] referred to with zones of inhibition ranging from 13 to 22 mm at 50 μg/disk. The fungus *Glarea lozoyensis* was isolated from pond water in the valley of the Lozoya River, Spain, and is capable of producing lipopeptide antibiotics pneumocandin A_0_ (**268**) and B_0_ (**269**) (Figure 26) [62]. The MIC value of pneumocandin B_0_ (**269**) against the fungal pathogen *Candida albicans* MY1208 was 0.06 µg/mL [63]. This compound could destroy the integrity of the cell wall of *C. albicans* by specifically inhibiting the synthesis of 1,3–*β*–D–glucan in the cell wall, resulting in the osmotic pressure inside the fungal cell being unstable, and ultimately leading to the lysis of the fungal cell [63]. El–Elimat et al. [33] obtained two known cyclodepsipeptides, Sch 378161 (**270**) and Sch 217048 (**271**) (Figure 26), from the fungi of *Clohesyomyces aquaticus* G100 and *Clohesyomyces* sp. G102 is associated with submerged wood from North Carolina, USA. However, both compounds were inactive against the bacteria of *Staphylococcus aureus* and *Escherichia coli* and the fungi of *Mycobacterium smegmatis*, *Candida albicans*, and *Aspergillus niger*.

**Figure 27:**
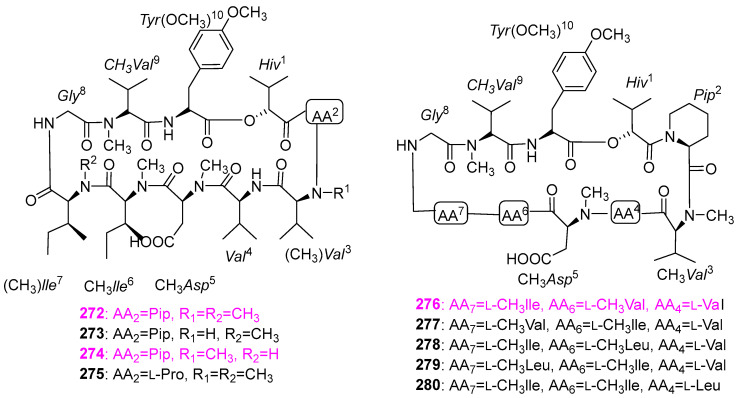
Chemical structures of compounds **272**–**280** from *Clavariopsis aquatica*.

The scale fermentation of the freshwater–derived hyphomycete *Clavariopsis aquatica* (AJ117363), which was collected from alpine mountain streams in Tokyo, Japan, afforded two known homologous clavariopsins A (**272**) and B (**273**) and seven new cyclic depsipeptides, clavariopsins C–I (**274**–**280**) (Figure 27) [64]. These nine compounds were tested for the inhibitory effect on the growth of six plant pathogens, including *Botrytis cinerea* NBc1, *Magnaporthe oryzae* Ken53–35, *Colletotrichum orbiculare* 104–T, *Fusarium oxysporum* CK3–1, *Alternaria alternata* M–71, and *Aspergillus niger* AJ117065. Specifically, all of the clavariopsins had significant or moderate activity against two important plant pathogenic fungi of *B. cinerea* NBc1 and *A. alternata* M–71, with MID values ranging from 0.01 to 0.3 μg/disk. Among them, clavariopsins A (**272**), C (**274**), and D (**276**) showed the highest activity against *A. alternate* M–71 with an MID value of 0.01 μg/disk. In addition, it was observed that all of the isolated compounds could induce hyphal malformation of *A. niger* AJ117065 at the MID value of 0.3 μg/disk for **272** and **274**–**279** and at 3 μg/disk for **273** and **280**, respectively. The results of antifungal activity suggested that this class of clavariopsins should contain potential natural fungicides. Whereas, none of compounds **272**–**280** exhibited significant cytotoxic against HeLa–S3 cells at the concentration of 10 μM.

### 2.12. Terpenoids

**Figure 28:**
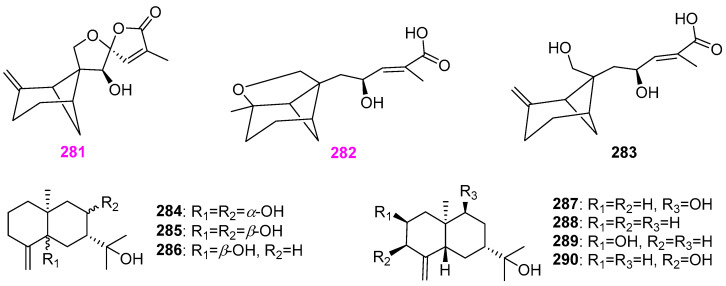
Chemical structures of compounds **281**–**283** from *Massarina tunicata*, and **284**–**290** from *Beltrania rhombica*.

Three new massarinolins A–C (**281**–**283**) (Figure 28) [65], as the first report of secondary metabolites from any member of the genus *Massarina*, were discovered in the EtOAc crude extract of filtered fermentation broth of the mentioned fungus *Massarina tunicata* A–25–1 producing furanones **25**–**28** (Figure 2) and pyrones **45** and **46** (Figure 4) [25]. These new sesquiterpenoids possess unusual tetracyclic and tricyclic ring systems. At 200 µg/disk, compounds **281** and **282** showed activity against *Bacillus subtilis* (ATCC 6051), causing zones of inhibition of 17 and 8 mm, and compound **281** displayed activity against *Staphylococcus aureus* (ATCC 29213), affording a 10 mm zone of inhibition. The freshwater fungus *Beltrania rhombica* (strain T031) was collected from the Ton–Nga–Chang Wildlife Sanctuary, Songkhla Province in southern Thailand [66]. The EtOAc crude extract of the culture broth of the fungal strain T031 exhibited potent antimicrobial activity against the bacterium *Staphylococcus aureus* (ATCC 25923) and clinical isolates of *Candida albicans* with MIC values of 0.98 and 15.6 mg/mL, respectively. The chemical investigation of secondary metabolites of strain T031 gave two new sesquiterpenes, rhombitriol (**284**) and rhombidiol (**287**), and five known ones, **285**, **286**, and **288**–**290** (Figure 28). All nine compounds showed weak activity against the above pathogens with MIC values ˃ 128 mg/mL.

**Figure 29:**
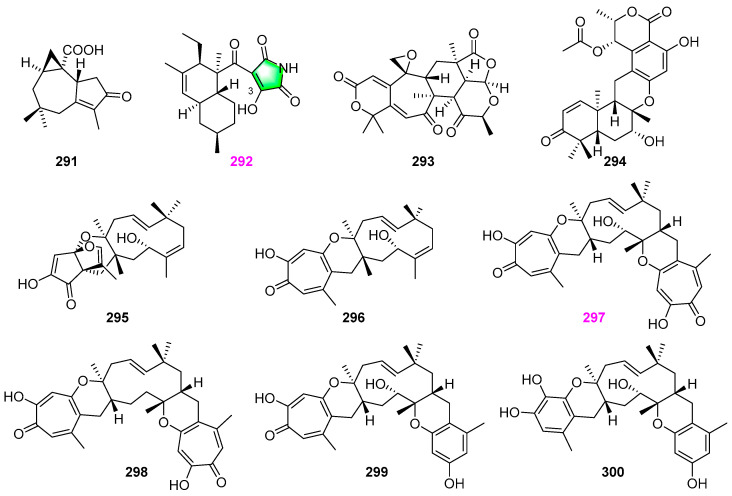
Chemical structures of compounds **291** from *Ophioceras venezuelense*, **292** from *Vaginatispora aquatica*, **293** and **294** from *Talaromyces amestolkiae*, and **295**–**300** from *Neosetophoma* sp. The green highlight indicates an important pharmacophore.

A new africane sesquiterpenoid ophioceric acid (**291**) (Figure 29) and the mentioned above four novel tetrahydropyran derivatives **32**–**35** (Figure 3) were obtained from the aquatic fungus *Ophioceras venezuelense* (strain A447–1B) [28]. The new compound **291** was also inactive toward *Candida albicans*, *Staphylococcus aureus*, and *Escherichia coli* at 200 µg/disk. He et al. [16] found that the fungal strain of *Vaginatispora aquatica* HK1821 from a decaying piece of wood submerged in the Lam Tsuen River in Tai Po of Hong Kong could produce the new compound oxasetin (**292**) (Figure 29) belonging to 2–oxo–succinimide polyketide. Although the 2–oxo–succinimide is usually reported as an important pharmacophore in many chemosynthetic bioactive compounds [67], the new compound oxasetin (**292**) is the first example of a natural product bearing this moiety. Compound **292** exhibited moderate antibacterial activity against gram–positive bacteria, methicillin–resistant *Staphylococcus aureus*, and vancomycin–resistant *Enterococcus faecalis*, with MIC values ranging from 16 to 32 μg/mL, but no activity against gram–negative bacterium *Escherichia coli* and the yeast *Candida albicans*. El–Elimat et al. [34] obtained a known meroterpenoid berkeleyacetal C (**293**) and a new merodrimane *ent*–thailandolide B (**294**) (Figure 29) besides the above pyranone derivatives **94**–**100** (Figure 7) from the fungus *Talaromyces amestolkiae* (strain G173). Both compounds **293** and **294** showed neither antibacterial nor antifungal activity against the five pathogens mentioned above. Five new metabolites—neosetophomone A (**295**) with a dioxa[4.3.3]propellane ring system, neosetophomone B (**296**), eupenifeldin (**297**), dehydroxyeupenifeldin (**298**), noreupenifeldin B (**299**), and one known 22–hydroxyramiferin (**300**) (Figure 29) were isolated from *Neosetophoma* sp. (strain MSX50044) [68]. The structure of compound **296** was further verified via single–crystal X–ray diffraction, and the absolute configurations of compounds **295**–**297** were confirmed via Mosher’s ester method, modified Mosher’s ester method, and vibrational circular dichroism (VCD), respectively. Compounds **295**–**300** were evaluated against six cell lines, including MDA−MB−231 (human breast cancer), OVCAR–3, and OVCAR–8 (human ovarian cancers), MSTO211H (human mesothelioma), LLC (murine lung cancer), and A549 (human lung cancer). Among them, meroterpenoids **297**–**299** showed excellent cytotoxicity against breast, ovarian, mesothelioma, and lung cancer cells (IC_50_ values ranging from 7.06 to 0.01 μM), while not inducing mitochondrial toxicity at 12.5 μM. Compound **297** was considered a potent cytotoxic agent with IC_50_ values of 2830, 330, 20, 80, 10, and 1330 nM, respectively.

### 2.13. Steroids

**Figure 30:**
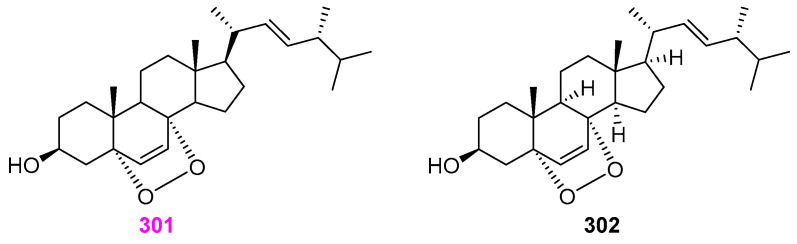
Chemical structures of compounds **301** from an unidentified fungi YMF 1.01029, and **302** from *Lindgomyces angustiascus*.

Besides pyran analogs **36**–**38** (Figure 3) and twelve naphthalene and naphthalenone derivatives **175**–**186** (Figure 15) as stated previously, Dong et al. [48] also obtained 3*β*–hydroxy–5*α*,8*α*–epidioxyergosta–6,22–diene (**301**) from the unidentified aquatic fungal isolate YMF 1.01029 (Figure 30). This steroid compound had no antimicrobial activity against *Bacillus cereus* YMF 3.19 and *Fursarium verticillioides* YMF 1.2076. Raja et al. [17] obtained ergosterol peroxide compound **302** (Figure 30), besides a fatty acid **1**, as previously shown in Figure 1, from the fungus *Lindgomyces angustiascus* (G202–1). There was no report of bioactivity for compound **302**.

### 2.14. Other Compounds

**Figure 31:**
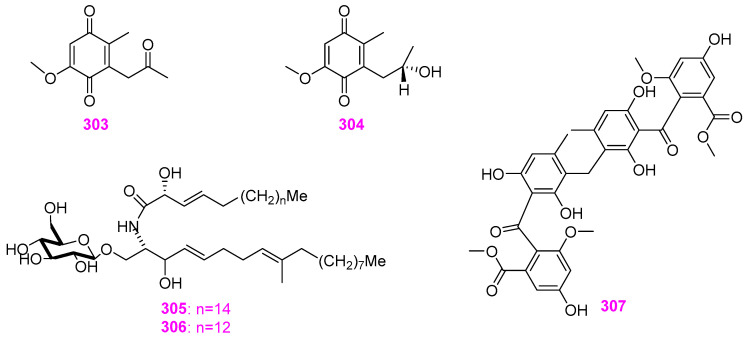
Chemical structures of compounds **303** and **304** from *Penicillium* sp., **305** and **306** from *Para niesslia* sp., and **307** from possibly *Entrophospora* sp. and *Phaeosphaeria* sp.

The earlier mentioned fungus *Penicillium* sp. (S1a1) was demonstrated to produce pyran isochromans **41**–**43** (Figure 4), tanzawaic acid derivatives **166**–**174** (Figure 14), and anserinones A (**303**) and B (**304**), as shown in Figure 30 herein [31]. Both of the isolated metabolites, **303** and **304**, showed moderate cytotoxicity activity against the mouse lymphoma L5178Y cell line with IC_50_ values of 27.4 and 20.9 μM, respectively, while compound **304** exhibited weak antibacterial activity against two strains (ATCC 700699 and ATCC 29213) of *Staphylococcus aureus* with an MIC value of 50 µM. A new (2*S*,2′*R*,3*R*,3′*E*,4*E*,8*E*)–1–*O*–*β*–D–glucopyranosyl)–3–hydroxyl–2–[*N*–2′–hydroxyl–3′–eicosadecenoyl]amino–9–methyl–4,8–octadecadiene (**305**) and a known compound **306** (Figure 31) were isolated from the mycelial cultures of the aquatic fungus *Paraniesslia* sp. YMF1.01400 derived from a woody substrate submerged in Lake Fuxian, Yunnan, China [69]. Both compounds showed identically killing activity against *Bursaphelenchus xylophilus* with LC_50_ values of 110 µg/mL. Two fungi (possibly *Entrophospora* sp. and *Phaeosphaeria* sp.) could produce disulochrin (**307**) (Figure 31) along with furanones **26** and **27** (Figure 2) and pyranones **77**–**79** (Figure 6) previously described [27]. Compound **307** was observed to completely inhibit the growth of methicillin–resistant *Staphylococcus aureus* (MRSA) (ATCC 700787) at the concentration of 100 µg/mL, but exhibited no activity against *Klebsiella pneumoniae* ATCC 51503, polyene–resistant *Candida albicans* ATCC 38245, and *Aspergillus fumigatus* FGSC A1100.

## 3. Systematic Review and Trend Analysis of Secondary Metabolites of Freshwater Fungi

**Figure 32:**
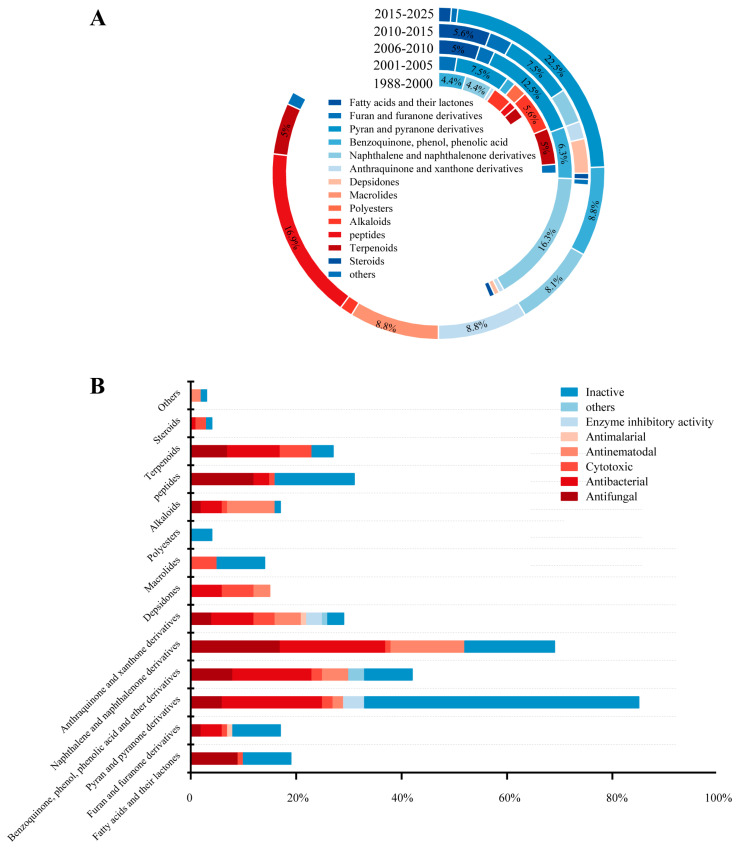
(**A**) Statistics on natural products isolated from freshwater fungi from 1988–2025; (**B**) The proportion of each of several classes of compounds with various biological activities.

**Figure 33:**
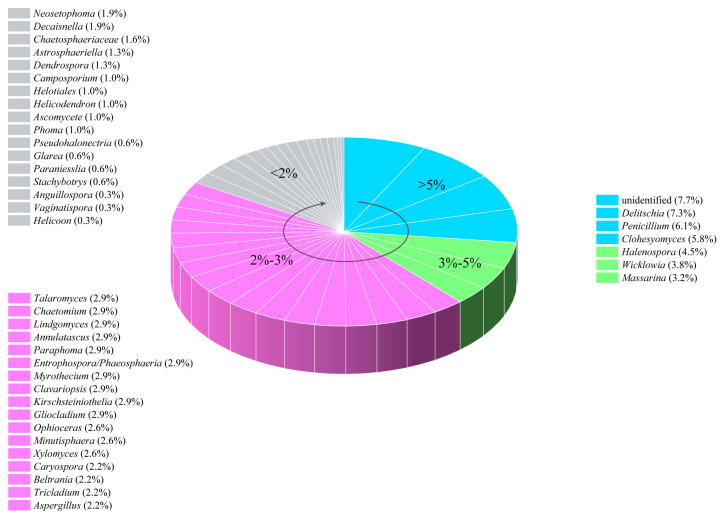
The percentage of the total isolated secondary metabolites from freshwater fungi. The blue sector denotes fungal genera whose natural products account for over 5% of the total, the pink sector indicates those whose natural products constitute between 2% and 5% of the total, while the grey sector represents fungal genera whose natural products constitute less than 2% of the total.

Since the publication of the first article exploring the metabolites of freshwater fungi in 1988 [10], research on freshwater fungi has been continuously developed. As shown in Figure 32A, a total of 307 compounds, including fatty acids and their lactones (5.86%), furans and furanones (4.23%), pyrans and pyranones (25.41%), benzoquinones, phenols and phenolic acids (10.42%), naphthalenes and naphthalenones (16.61%), and authraquinones and xanthones (6.19%), depsidones (1.95%), macrolides (5.54%), polyesters (0.98%), alkaloids (4.56%), peptides (9.45%), terpenoids (6.51%), and steroids (0.65%), and other compounds (1.63%) have been isolated and characterized from 41 different freshwater fungal genera and unidentified taxa (Table 1). Analysis of the discovery trends reveals a rapid increase in the number of metabolites from freshwater fungi over time (Figure 32A), with notable growth in the past decade for furan and furanone derivatives and terpenoids. However, when viewed in a broader context, among the newly reported 1160 fungal natural products in the last two years, approximately two–thirds originated from plant–associated fungi (38%) and marine–derived fungi (30%), with freshwater fungi contributing only a minor fraction. This highlights that freshwater fungi remain a relatively underexplored resource. Nevertheless, existing studies have clearly demonstrated their significant potential. Secondary metabolites derived from these fungi exhibit mainly antimicrobial, cytotoxic, enzyme inhibitory, and anti–nematode activities (Figure 32B). Notably, new compounds account for 47.3% of the total, among which 146 compounds possess novel skeletons. This collectively underscores the considerable value of freshwater fungi as a “reservoir” for novel natural products. Further analysis of the sources of active metabolites found that the fungal genera of *Delitschia*, *Penicillium*, and *Clohesyomyces* were the main sources of bioactive agents (Figure 33, Table 1).

## 4. Conclusions and Looking Ahead

The summarized results of chemical investigation and biological evaluation for these 307 compounds in this review indicate that natural products from freshwater fungi have potent chemical diversity, and those with excellent bioactivities may be considered as important candidates for the development of antibiotics, anticarcinogens, and insecticides in the field of medicine and agriculture. Nevertheless, as shown in Figure 32B, there are still a number of compounds that are inactive in the above–mentioned bioassays or have not screened for other bioactivities. Therefore, in order to better exploit the bioactive potential of natural products from freshwater fungi, more bioactive models should be established and applied to the screening of the biological activity of compounds. Moreover, it cannot be ignored that nearly all the highly active natural products obtained from freshwater fungi over the past decades have not been referred to in further research on their mechanisms of action or toxicities, according to our knowledge, not to mention medicinal and industrial application studies. This suggests that the low yield of some potent bioactive secondary metabolites still remains as a major stumbling block in their future development. As highlighted in previous reviews of natural products from microbes [1,70], genomic technologies can be employed to generate sufficient quantities of target compounds and discover new biosynthetic gene clusters. Last but not least, fungi inhabiting special environments have been increasingly investigated to discover new potential bioactive compounds [71]. Recently, Shen et al. [72] and Luo et al. [73] have found that the lignicolous fungi isolated from plateau lakes in Yunnan province of China displayed good species diversity and novelty, which means that these fungi may have potent developing value as a biological resource. Therefore, focusing on the exploitation of microbes in special ecosystems, such as plateaus and glaciers, salt marshes, hot spring and so on, may be significant for rational utilization of characteristic and rare microorganisms in special environments and their secondary metabolites.

## Figures and Tables

**Table 1:** The producing strain (genus), compounds, biological activity, and type of compounds of freshwater fungi.

Producing Strain (Genus)	Compounds	Biological Activity	Type of Compounds	Refs.
*Lindgomyces*	6*E*,9*E*–octadecadienoic acid (**1**)	/	Fatty acids and their lactones	[17]
	Ergosterol peroxide (**302**)	/	Steroids	
*Minutisphaera*	Palmitic acid (**2**)	/	Fatty acids and their lactones	[18]
	(*E*)–hexadec–9–en–1–ol (**3**)	/		
*Tricladium*	Tricladic acids A (**4**)	/	Fatty acids and their lactones	[19]
	Tricladic acids B and C (**5** and **6**)	Antifungal		
	Tricladolides A–C (**7**–**9**)	Antifungal		
	Tricladolide D (**10**)	Antifungal; cytotoxic		
*Wicklowia*	Tetrahydroepiheveadride (**11**)	/	Fatty acids and their lactones	[20]
	Dideoxoepiheveadride (**12**)	/		
	Deoxodihydroepiheveadride (**13**)	/		
	Epiheveadride (**14**)	Antifungal		
	Deoxoepiheveadride (**15**)	Antifungal		
	Dihydroepiheveadride (**16**)	Antifungal		
	Waquafranones A and B (**17** and **18**)	/		
	Sydonic acid (**135**)	/	Benzoquinone, phenol, phenolic acid, and their derivatives	
	Hydroxysydonic acid (**136**)	/		
	Agonodepside B (**137**)	/		
	Folipastatin (**138**)	/		
*Helicodendron*	Heliconol A (**19**)	Antibacterial; antifungal	Furan and furanone derivatives	[21]
	Heliconols B and C (**20** and **21**)	/		
*Chaetosphaeriaceae*	Mansonone D (**22**)	Antimalarial; antifungal; cytotoxicity	Furan and furanone derivatives	[22]
	Xanthones I and III (**198** and **200**)	/	Anthraquinone and xanthone derivatives	
	Xanthone II (**199**)	Antimalarial; Antibacterial; antifungal; cytotoxicity		
	Xanthone IV (**201**)	Antibacterial; antifungal; cytotoxicity		
*Annulatascus*	Annularins G and H (**23** and **24**)	/	Furan and furanone derivatives	[24]
	Annularins D and E (**62** and **63**)	/	Pyran and pyranone derivatives	
	Annularins A–C, F (**59**–**61**, **64**)	Antibacterial		
	(−)–(*S*)–*p*–hydroxyphenyllactic acid (**111**)	/	Benzoquinone, phenol, phenolic acid, and their derivatives	
*Massarina*	Massarigenins A, C, D (**25**, **27**, **28**)	Antibacterial	Furan and furanone derivatives	[25]
	Massarigenin B (**26**)	/		
	Massarinins A and B (**45** and **46**)	Antibacterial	Pyran and pyranone derivatives	
	4–(2–hydroxybutynoxy) benzoic acid (**113**)	Antibacterial; antifungal	Benzoquinone, phenol, phenolic acid, and their derivatives	
*Paraphoma*	(*R*)–7–hydroxy–3–((*S*)–1–hydroxy–ethyl)–5–methoxy–3,4–dimethylisobenzofuran–1(3*H*)–one (**29**)	/	Furan and furanone derivatives	[26]
	(*R*)–3,4–dihydro–4,6,8–trihydroxy–4,5– dimethyl–3–methyleneisochromen–1–one (**88**)	/	Pyran and pyranone derivatives	
	(*R*)–3,4–dihydro–4,8–dihydroxy–6–methoxy–4,5– dimethyl–3–methyleneisochromen–1–one (**89**)	/		
	3,8–dihydroxy–3–hydroxymethyl–6–methoxy– 4,5–dimethyl–isochroman–1–one (**92**)	/		
	Clearanol C (**90**)	Antibacterial		
	Clearanols F and G (**93** and **91**)	/		
	Isosclerone (**146**)	/	Naphthalene and naphthalenone derivatives	
	Radinaphthalenone (**147**)	/		
*Entrophospora* *Phaeosphaeria*	Clearanol E (**30** and **31**)	/	Furan and furanone derivatives	[27]
	Clearanols A–B, D (**77**–**79**)	/	Pyran and pyranone derivatives	
	Disulochrin (**307**)	/	Others	
*Ophioceras*	Ophiocerins A–D (**32**–**35**)	/	Pyran and pyranone derivatives	[28]
	Ophioceric acid (**291**)	/	Terpenoids	
unidentified	Colomitide A (**36**)	Antibacterial; antifungal	Pyran and pyranone derivatives	[29]
	Colomitide B (**37**)	Antibacterial; antifungal		
	Preussomerin E (**186**)	Antibacterial; antifungal	Naphthalene and naphthalenone derivatives	
unidentified	(2*RS*,2′*SR*,3′*E*,3*SR*,4*E*,8*E*)–1–*O*–(*β*–D–glucopyranosyl)– 3–hydroxy–2–[(2–hydroxyoctadec–3–enoyl) amino]– 9–methyloctadeca–4,8–diene (**38**)	/	Pyran and pyranone derivatives	[29,48,49]
	Colelomycerone A (**177**)	Antibacterial; antifungal	Naphthalene and naphthalenone derivatives	
	Colelomycerone B (**178**)	Antibacterial; antifungal		
	Preussomerin D (**185**)	Antibacterial; antifungal; Antinematode activities		
	3*β*–hydroxy–5*α*,8*α*–epidioxyergosta–6,22–diene (**301**)	/	Steroids	
*Pseudohalonectria*	Pseudohalonectrins A and B (**39** and **40**)	Antinematode activities	Pyran and pyranone derivatives	[30]
*Penicillium*	(3*S*)–6–hydroxy–8–methoxy– 3,5–dimethylisochroman (**41**)		Pyran and pyranone derivatives	[31]
	(3*S*,4*R*)–6–hydroxy–8–methoxy– 3,5–dimethylisochromanol (**42**)	/		
	(1*S*,3*S*)–1,6–dihydroxy–3,5– dimethyl–8–methoxyisochroman (**43**)	/		
	Penitanzchroman (**166**)	/	Naphthalene and naphthalenone derivatives	
	Tanzawaic acids Y, Z, A, E, M, N (**167**, **168**, **170**, **172**–**174**)	/		
	Arohynapene A (**169**)	/		
	Tanzawaic acid B (**171**)	Antibacterial		
	Anserinone A (**303**)	Cytotoxicity	Others	
	Anserinone B (**304**)	Antibacterial; cytotoxicity		
*Delitschia*	(3S*,4S*,5S*,6R*)–4,5,6–trihydroxy–3–methyl– 3,4,6,7–tetrahydro–1*H*–isochromen–8(5*H*)–one (**44**)	Antibacterial; antifungal	Pyran and pyranone derivatives	[12]
	(3R*,4S*)–7–ethyl–3,4,6,8–tetrahydroxy– 3,4–dihydronaphthalen–1–(2*H*)–one (**150**)	Antibacterial; antifungal	Naphthalene and naphthalenone derivatives	
	6–ethyl–7–hydroxyl–2–methoxyjuglone (**151**)	Antibacterial; antifungal		
	6–ethyl–2,7–dimethoxyjuglone (**152**)	Antibacterial; antifungal		
	6–(1–hydroxyethyl)–2,7–dimethoxyjuglone (**153**)	Antibacterial; antifungal		
	Sporidesmin A (**265**)	Antibacterial; antifungal	peptides	
*Delitschia*	Delitpyrone A (**47**)	/	Pyran and pyranone derivatives	[32]
	1′,2′–epoxi–delitpyrone A (**48**)	Cytotoxic		
	Delitpyrones B, C D (**49**, **50**, **52**)	/		
	2′–oxodelitpyrone A (**51**)	/		
	5–(2–hydroxybutyl)–4–methoxy–6–methyl–2*H*–pyran–2one (**53**)	/		
	5–(3–*S*–hydroxybutyl)–4–methoxy–6–methyl–2*H*–pyran–2–one (**54**)	/		
	5–(3–oxobutyl)–4–methoxy–6–methyl–2*H*–pyran–2–one (**55**)	/		
	Pyrenocine I (**56**)	/		
	3′–hydroxydelitpyrone D (**57**)	/		
	5–butyl–6–(hydroxymethyl)–4 –methoxy–2*H*–pyran–2–one (**58**)	/		
	3S*,4S*–7–ethyl–4,8–dihydroxy–3,6–dimethoxy– 3,4–dihydronaphthalen–1(2*H*)–one (**148**)	/	Naphthalene and naphthalenone derivatives	
	6–ethyl–2,7–dimethoxyjuglone (**149**)	Cytotoxic		
	Sporidesmin A (**265**)	Cytotoxic	peptides	
	Artrichitin (**266**)	/		
	lipopeptide 15G256ε (**267**)	/		
*Clohesyomyces*	Phomopsinones A and B (**65** and **68**)	Enzyme inhibitory activity	Pyran and pyranone derivatives	[33]
	Pyrenocines K and M (**66** and **71**)	/		
	Pyrenocine P (**67**)	Enzyme inhibitory activity		
	Phomopsinone C (**69**)	/		
	6–hydroxy–7–*epi*–phomopsinone A (**70**)	Enzyme inhibitory activity		
	5–deoxy–7–hyrdoxypyrenocine M (**72**)	/		
	7–hyrdoxypyrenocine M (**73**)			
	Pyrenocines Q and R (**74** and **75**)	/		
	5–hydroxyphomopsinone A (**76**)	/		
	8–hydroxyblennolide H (**202**)	Enzyme inhibitory activity	Anthraquinone and xanthone derivatives	
	*cis*–dihydro–8–hydroxyblennolide H (**203**)	Enzyme inhibitory activity		
	*trans*–dihydro–8–hydroxyblennolide H (**204**)	/		
	Secalonic acid A (**205**)	Enzyme inhibitory activity		
	Sch 378161 (**270**)	/	Peptides	
	Sch 217048 (**271**)	/		
*Xylomyces*	(2*S*, 3*S*, 4*S*)–3,4–dihydroxy–2–methyl–7–propyl–3,4–dihydro–2*H*,5*H*–pyrano[4,3–*b*]pyran–5–one (**80**)	/	Pyran and pyranone derivatives	[13]
	(2*S*, 3*S*)–3,7–dihydroxy–2,5–dimethyl–chroman–4–one (**81**)	/		
	3–*epi*–radicinol (**82**)	/		
	Radicinol (**83**)	/		
	4–*epi*–radicinol (**84**)	/		
	3–*epi*–radicinol epoxide (**85**)	/		
	Radicinin (**86**)	Antifungal		
	3–*epi*–radicinin (**87**)	Antifungal		
*Talaromyces*	Aspergillumarins A and B (**94** and **95**)	/	Pyran and pyranone derivatives	[34]
	4–hydroxyaspergillumarin A (**96**)	/		
	3–hydroxymethyl–6,8–dimethoxycoumarin (**97**)	/		
	Pestalasin A (**98**)	/		
	7–chloropestalasin A (**99**)	/		
	Graphislactone A (**100**)	/		
	Berkeleyacetal C (**293**)	/	Terpenoids	
	*ent*–thailandolide B (**294**)	/		
unidentified	Dihydroaltenuene A (**101**)	Antibacterial	Pyran and pyranone derivatives	[35]
	Dihydroaltenuene B (**102**)	/		
	Dehydroaltenuenes A–B (**103**–**104**)	Antibacterial		
	Isoaltenuene (**105**)	Antibacterial		
	Altenuene (**106**)	Antibacterial		
	5′–epialtenuene (**107**)	/		
*Penicillium*	3–(furan 12–carboxylic acid)–6–(methoxycarbonyl)– 4–hydroxy–4–methyl–4, 5–dihydro–2*H*–pyran (**108**)	Antibacterial	Pyran and pyranone derivatives	[14]
	Austinol (**109**)	Antibacterial; cytotoxicity		
	3*α*–methyl–7–hydroxy–5– carboxylic acid methyl ester–1–indanone (**114**)	Antibacterial; cytotoxicity	Benzoquinone, phenol, phenolic acid, and their derivatives	
	Emodin (**193**)	Antibacterial; cytotoxicity	Anthraquinone and xanthone derivatives	
	2–methyl–penicinoline (**238**)	Antibacterial	Alkaloids	
*Camposporium*	2′,4′–dihydroxyacetophenone (**110**)	Antibacterial	Benzoquinone, phenol, phenolic acid, and their derivatives	[36]
	Tenellic acid A (**125**)	Antibacterial		
	Quercilolin (**132**)	Antibacterial		
*Anguillospora*	2,4–dihydroxy–3–ethyl–6– (1′–methylpentyl)–benzaldehyde (**112**)	Antibacterial; antifungal	Benzoquinone, phenol, phenolic acid, and their derivatives	[37]
*Ophioceras*	Caffeic acid (**115**)	Antifungal; antinematode activities	Benzoquinone, phenol, phenolic acid, and their derivatives	[38]
	Isoamericanoic acid A (**116**)	Antifungal; antinematode activities		
	Ophiocerol (**117**)	Antifungal		
*Lindgomyces*	Madisone (**118**)	Antibacterial; Antifungal;	Benzoquinone, phenol, phenolic acid, and their derivatives	[39]
	4′–methoxymadisone (**119**)	Antibacterial; Antifungal;		
	Dehydromadisone (**120**)	/		
	2″–methoxymadisone (**121**)	/		
	Dihydroallovisnaginone (**122**)	Antibacterial; Antifungal		
	Dimadisone (**123**)	/		
	4′–methoxydimadisone (**124**)	/		
*Dendrospora*	Tenellic acids A–D (**125**–**128**)	Antibacterial	Benzoquinone, phenol, phenolic acid, and their derivatives	[40]
*Phoma*	1–methoxy–3,5′–dimethyl–2,3′– oxybiphenyl–5,1′,2′–triol (**129**)	Enzyme inhibitory activity	Benzoquinone, phenol, phenolic acid, and their derivatives	[41]
	5–methoxy–3,5′–dimethyl– 2,3′–oxybiphenyl–1,1′,2′–triol (**130**)	Enzyme inhibitory activity		
	Cyperine (**131**)	Enzyme inhibitory activity; cytotoxicity		
*Kirschsteiniothelia*	2,4–dichloro–3–(2–chloro–3–hydroxy–5–methylphenoxy)–5–methylphenol (**133**)	Antibacterial	Benzoquinone, phenol, phenolic acid, and their derivatives	[42]
	3,3′–oxybis(2,4–dichloro–5–methylphenol) (**134**)	Antibacterial		
	6–(1–hydroxyethyl)–2,7–dimethoxyjuglone (**159**)	/	Naphthalene and naphthalenone derivatives	
	1–(1–hydroxy–3,6–dimethoxy–5,8–dioxo–5,8–dihydronaphthalen–2–yl) ethyl acetate (**160**)	/		
	6–ethyl–7–hydroxy–2–methoxyjuglone (**161**)	/		
	6–ethyl–2,7–dimethoxyyjuglone (**162**)	/		
	(−)–*O*–methylasparvenone (**163**)	/		
	Kirschsteinin (**164**)	Cytotoxicity; antibacterial		
	ethylidene–6,6′–biplumbagin (**165**)	/		
*Helotiales*	Leotiomycenes A–C (**139**–**141**)	Suppress quorum sensing	Benzoquinone, phenol, phenolic acid, and their derivatives	[43]
*Caryospora*	4,8–dihydroxy–3,4–dihydronaphthalen–1(2*H*)–one (**142**)	Antinematode activities	Naphthalene and naphthalenone derivatives	[44,45]
	4,6–dihydroxy–3,4–dihydronaphthalen– 1(2*H*)–one (**143**)	Antinematode activities		
	4,6,8–trihydroxy–3,4– dihydronaphthalen–1 (2*H*) –one (**144**)	Antinematode activities		
	3,4,6,8– tetrahydroxy–3,4–dihydronaphthalen–1 (2*H*) –one (*cis*–4–hydroxyscytalone) (**145**)	Antinematode activities		
	Caryospomycins A–C (**218**–**220**)	Antinematode activities	Depsidones	
*Astrosphaeriella*	Astropaquinones A–C (**154**–**156**)	Antibacterial; antifungal	Naphthalene and naphthalenone derivatives	[46]
	6–hydroxy–2,4–dimethoxy–7–methylanthraquinone (**194**)	Antibacterial; antifungal	Anthraquinone and xanthone derivatives	
*Minutisphaera*	Isosclerone (**157**)	/	Naphthalene and naphthalenone derivatives	[47]
	Sphaerolone (**158**)	Antibacterial		
	Cyclo–([*S*]–Pro–[*S*]–Leu) (**252**)	/	peptides	
	Cyclo–([*R*]–Pro–[*S*]–Phe) (**253**)	–/		
	Cyclo–([*S*]–Pro–[*S*]–Phe (**254**)	/		
	Cyclo–([*S*]–Ala–[*S*]–Phe) (**255**)	/		
unidentified	(4*RS*)–4,8–dihydroxy–3,4–dihydronaphthalen–1(2*H*)–one (**175**)	Antinematode activities	Naphthalene and naphthalenone derivatives	[29,48,49]
	4,6,8–trihydroxy–3,4–dihydronaphthalen–1(2*H*)– one (**176**)	Antinematode activities		
	ymf 1029A–E (**179**–**183**)	Antinematode activities		
	Preussomerins C and D (**184** and **185**)	Antinematode activities		
*Decaisnella*	Decaspirones A–E (**187**, **190**, **191**, **188**, **189**)	Antibacterial; antifungal	Naphthalene and naphthalenone derivatives	[50]
	Palmarumycin CP1 (**192**)	Antibacterial; antifungal		
*Chaetomium*	Citreorosein (**195**)	Antibacterial	Anthraquinone and xanthone derivatives	[51]
	Emodin (**196**)	Antibacterial		
	1–hydroxy–6–methyl–8–hydroxymethyl–xanthone (**197**)	Cytotoxicity; antibacterial		
	Chaetones A–F (**212**–**217**)	Cytotoxicity; antibacterial	Depsidones	
*Helicoon*	Quinaphthin (**206**)	Antifungal; antibacterial Anti–arichomonas vaginalis	Anthraquinone and xanthone derivatives	[10]
*Aspergillus*	Asperdichrome (**207**)	Enzyme inhibitory activity	Anthraquinone and xanthone derivatives	[53]
	Secalonic acid D (**208**)	Enzyme inhibitory activity		
	Secalonic acid F (**209**)	Enzyme inhibitory activity		
	Secalonic acid F (**210**)	Enzyme inhibitory activity		
	Secalonic acid F1 (**211**)	Enzyme inhibitory activity		
*Halenospora*	Greensporone A (**221**)	Cytotoxic	Macrolides	[54]
	Greensporone C (**223**)	Cytotoxic		
	Greensporones B, D–G (**222**, **224**–**227**)	/		
	8,9–dihydrogreensporones A, C, D (**228**, **229**, **230**)	/		
	Dechlorogreensporones A and D (**231** and **232**)	Cytotoxic		
	Dechlorogreensporone F (**233**)	/		
	*O*–desmethylgreensporone C (**234**)	Cytotoxic		
*Ascomycete*	W1278–A–C (**235–237**)	/	Polyesters	[57]
*Stachybotrys*	Stachybotrin A (**239**)	Antibacterial; antifungal Cytotoxicity	Alkaloids	[58]
	Stachybotrin B (**240**)	Antibacterial; antifungal		
*Aspergillus*	Speramide A (**241**)	Antibacterial	Alkaloids	[15]
	Speramide B (**242**)	/		
*Paraniesslia*	(2*S*,2′*R*,3*R*,3′*E*,4*E*,8*E*)–1–*O*–(*β*–D–glucopyranosyl)– 3–hydroxyl–2–[N–2′–hydroxyl–3′–eicosadecenoyl]amino–9–methyl–4,8–octadecadiene (**305**)	Antinematode activities	Others	[69]
	(2*S*,2′*R*,3*R*,3′*E*,4*E*,8*E*)–1–*O*–(*β*–D–glucopyranosyl)– 3–hydroxyl–2–[N–2′–hydroxyl–3′–octadecenoyl]amino–9–methyl–4,8–octadecadiene (**306**)	Antinematode activities		
*Gliocladium*	Gliocladines A–E (**243**–**247**)	Antinematode activities	Alkaloids	[59]
	Verticillin A (**248**)	Antinematode activities		
	11′–deoxyverticillin A (**249**)	Antinematode activities		
	Sch 52900 (**250**)	Antinematode activities		
	Sch 52901 (**251**)	Antinematode activities		
*Myrothecium*	7–hydroxy–3–methoxyviridicatin (**256**)	Antibacterial	Peptides	[61]
	Viridicatin (**257**)	Antibacterial		
	3–methoxyviridicatin (**258**)	/		
	Viridicatol (**259**)	/		
	(11a*S*)–2,3–dihydro–1*H*–pyrrolo[2,1–c]– [1,4] benzodiazepine–5,11(10*H*,11a*H*)–dione (**260**)	/		
	Cyclopeptine (**261**)	/		
	Cyclopenin (**262**)	/		
	Dehydrocyclopeptin (**263**)	/		
	trans–3–(3′–hydroxy–benzylidene)–3,4–dihydro– 4–methyl–l*H*–1,4–benzodiazepin–2,5–dione (**264**)	/		
*Vaginatispora*	Oxasetin (**292**)	Antibacterial	Terpenoids	[16]
*Glarea*	Pneumocandins A_0_–B_0_ (**268**–**269**)	Antifungal	peptides	[62]
*Clavariopsis*	Clavariopsins A–I (**272–274**, **276**–**279**, **275**, **280**)	Antifungal	peptides	[64]
*Massarina*	Massarinolins A and B (**281** and **282**)	Antibacterial	Terpenoids	[65]
	Massarinolin C (**283**)	/		
*Beltrania*	Rhombitriol (**284**)	Antibacterial; antifungal	Terpenoids	[66]
	(−)–*β*–eudesmol (**285**)	Antibacterial; antifungal		
	(−)–5*β*–hydroxy–*β*–eudesmol (**286**)	Antibacterial; antifungal		
	Rhombidiol (**287**)	Antibacterial; antifungal		
	(−)–pterocarpol (**288**)	Antibacterial; antifungal		
	(−)–chrysanthemol (**289**)	Antibacterial; antifungal		
	(−)–longilobol (**290**)	Antibacterial; antifungal		
*Neosetophoma*	Neosetophomones A and B (**295** and **296**)	Cytotoxicity	Terpenoids	[68]
	Eupenifeldin (**297**)	Cytotoxicity		
	Dehydroxyeupenifeldin (**298**)	Cytotoxicity		
	Noreupenifeldin B (**299**)	Cytotoxicity		
	22–hydroxyramiferin (**300**)	Cytotoxicity		

## Data Availability

All data are obtained from the references.
